# Custom-Designed Carbosilane-Based Reactive Surfactants for Surface Hydrophobization—AFM, Raman Microscopy, and XPS Investigations

**DOI:** 10.1021/acs.langmuir.6c05105

**Published:** 2026-10-29

**Authors:** Sylwia Ronka, Łukasz Lamch, Maciej Kuchowicz, Rafał Szukiewicz, Przemysław Szklarz, Krzysztof Gawarecki

**Affiliations:** † Department of Polymer Engineering and Technology, Faculty of Chemistry, 49567 Wrocław University of Science and Technology, Wybrzeże Wyspiańskiego 27, 50-370 Wrocław, Poland; ‡ Department of Engineering and Technology of Chemical Processes, Faculty of Chemistry, Wrocław University of Science and Technology, Wybrzeże Wyspiańskiego 27, 50-370 Wrocław, Poland; § Institute of Experimental Physics, Faculty of Physics, 49572 University of Wrocław, Maxa Borna 9, 50-204 Wroclaw, Poland; ∥ Faculty of Chemistry, 49572 University of Wrocław, F. Joliot-Curie 14, 50-383 Wrocław, Poland; ⊥ Institute of Theoretical Physics, Faculty of Fundamental Problems of Technology, Wrocław University of Science and Technology, Wybrzeże Wyspiańskiego 27, 50-370 Wrocław, Poland

## Abstract

The reactive carbosilane cationic surfactant (SK) has been studied for glass surface hydrophobization by blending with oligodimethylsiloxane derivatives, comprising epoxide (EO) and amine/alkoxyl motifs (AO), and deposition using three different approaches: spray, spin, and dip coating. Our contribution comprises the synthesis and structure characterization of a surfactant (^1^H NMR, FT-IR and Raman spectroscopies), the optimization studies for the layer deposition by tuning the chosen parameters and compositions, as well as the determination of contact angles and surface energies. The crucial roles of the layer composition and the deposition methodology were carefully investigated, especially in terms of the reactivity of SK with siloxane derivatives and glass surfaces. The experiments were supported by the calculation of solubility parameters (δ) and the dispersion (δ_D_), polar (δ_P_), and hydrogen bonding (δ_H_, including its splitting into donor (δ_HD_) and acceptor (δ_HA_) counterparts) components utilizing HSPiP software, showing the possibility of appropriate component blending. The layer structure and morphology were studied via atomic force microscopy (AFM) and X-ray photoelectron spectroscopy (XPS) analyses with Ar^+^ sputtering as well as in-depth Raman microscopy. It was demonstrated that spray coating was an inappropriate method since it provided an extremely rough layer, while dip coating provided a deposit of limited stability due to the presence of separated SK deposits. The optimal system comprised SK combined with AO by a spin-coating approach, enabling both surface hydrophobization and the formation of a stable, chemically attached layer on the glass surface.

## Graphical abstract

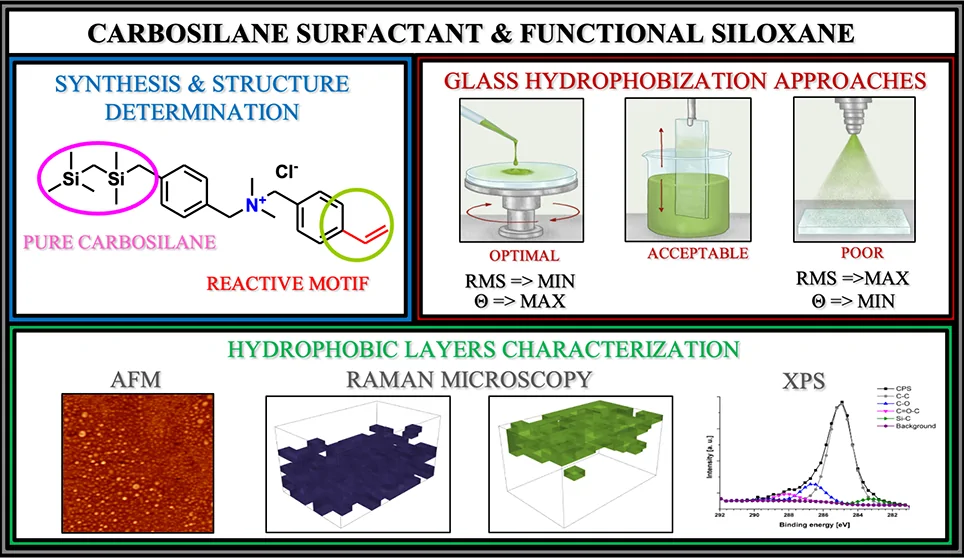

## Introduction

Silicone surfactants comprise a wide group of amphiphatic compounds, known for their unique properties and performance, especially in the field of very low minimal surface tension (even below 20 mJ/m^2^), activity in nonaqueous environments (e.g., polyurethane foam preformulations), and ease in expansion of the hydrophobic part into a hyperbranched structure.^1^ The majority of silicone surfactants comprise block or graft copolymers, consisting of poly­(dimethylsiloxane) and poly­(ethylene oxide)/poly­(propylene oxide) fragments, designed to fulfill specific requirements, especially in the field of flexible polyurethane foams. Such compounds, especially of high molecular weight, are rather hardly water-soluble and act as surfactants for organic-borne systems.^2^ The use of ionic silicone surfactants is limited, especially due to their susceptibility to undergo hydrolysis of Si–O–Si bonds, even under mild conditions (e.g., room temperature and slightly basic aqueous system), for low molecular weight compounds.^3^ One solution is the design and synthesis of carbosilane surfactants, comprising a hydrophobic moiety with linear or branched Si–C–Si motifs, significantly improving resistance to hydrolysis.^4^ Moreover, the introduction of a carbosilane motif instead of a typical siloxane one has an impact on the overall surfactant inter- and intramolecular behavior, including the chain packing and mobility.^3^ Such an approach, although opening new perspectives for silicone surfactants—especially in environments of high ionic strength values—performance, is not commonly studied.^5,6^ To the best of our knowledge, there are no examples of cationic carbosilane surfactants studied in the literature, in particular, comprising any reactive, e.g., characterized by ability to undergo polymerization, moiety.

The use of reactive (polymerizable), cationic surfactants toward surface hydrophobization is extremely rarely studied, taking into account the current state of the art.^7^ In general, only a few known compounds have been synthesized and characterized,^7–10^ although none of them has been mixed with commercially available functional polymers/oligomers to form a composition for modification of particular materials’ surface hydrophobicity. Silicones, especially derivatives of poly­(dimethylsiloxane), are very promising agents toward glass or silica surface hydrophobization.^7,11^ Typically, such a procedure involves physical and chemical adsorption of an appropriate siloxane derivative, often with reactive groups like hydroxyl or vinyl,^12^ especially at elevated temperatures (up to 250 °C).^11^ An elevated temperature enables even the reaction between poly­(dimethylsiloxane) without any hydrolyzable motifs and hydroxyl groups at the silica surface.^11^ Some of these reactions may be initiated by specific additives like dimethyl carbonate,^13^ involve ring-opening processes,^14^ or require pretreatment with a functional (epoxy) siloxane derivative.^15^ On the other hand, any heating is completely unacceptable for numerous applications, e.g., electronic devices, some composites, or materials obtained via controlled thermal treatment. Therefore, we focused on initial glass preparation methods acceptable for electronic devices, Au vapor depositions, etc.^16^ rather than harsh conditions like concentrated H_2_SO_4_, 1 M aqueous NaOH, or K_2_Cr_2_O_7_ + H_2_SO_4_.^17^ This initial step is crucial for any further hydrophobization step since it assumes total removal of impurities from the glass surface and its activation toward appropriate reactivity with silicones and surfactants.^17^

Hydrophobic layer characterization should specify surface homogeneity, wettability, and the presence of unreacted compounds. Moreover, the determination of contact angles and surface energies comprises the first crucial step toward layer characterization, since it shows if the layer is indeed hydrophobic, taking into account the macroscopic point of view.^18^ Contact angles and surface free energy (γs), determined using the Owens–Wendt–Rabel–Kaelble (OWRK) method, provide key information on surface wettability and interfacial properties.^18–20^ Surface morphology and roughness, which strongly influence wettability and reactivity, can be evaluated using techniques such as AFM and optical profilometry.^21,22^ Complementary information on surface composition and structure can be obtained by Raman spectroscopy and XPS. In particular, XPS combined with Ar^+^ sputtering enables elemental and chemical-state analysis as a function of depth.^23–30^ For organic coatings, this approach can be used to assess chemical homogeneity and changes in functional groups with depth.^28^ However, Ar^+^ sputtering may damage organic layers through bond breaking, preferential sputtering, and changes in chemical states, potentially introducing artifacts into the resulting depth profiles.^30,31^

The main aim of the present research was to investigate a new carbosilane reactive surfactant toward glass surface hydrophobization and determine the optimal composition and conditions for its deployment by spray, spin, and dip coating approaches. The particular points of interest comprise: (i) the use of different oligodimethylsiloxanes, mixed with the reactive silicone surfactant, for surface hydrophobization; (ii) optimization studies for three different methods toward hydrophobic layer deployment; (iii) the determination of contact angles and surface energies (dispersion and polar components) for the modified glass, as well as careful XPS and Raman microscopy studies of the selected (on the basis of AFM analysis with the determination of surface roughness) layers, characterized by superior performance. Our investigations aimed to carefully study the structure of the obtained organic layer and the zone of its interaction with glass by means of Raman microscopy and XPS with ion sputtering. The studies performed constitute an initial crucial step for a newly devised method of surface hydrophobization utilizing an engineered class of amphiphilic compounds—reactive carbosilane surfactants—capable of mixing and reacting with appropriate, commercially available oligodimethylsiloxane derivatives. Those oligomeric compounds were chosen for their possibility of chemical binding with the glass surface via covalent bonds (epoxide ring opening or alkoxyl motifs hydrolysis) or weaker interactions (interactions between amine groups and oxides/hydroxides at the surfaces). Our aim was to exploit both the reactive motifs from commercial siloxane derivatives (amine or epoxy) and the ability of our carbosilane surfactant to undergo polymerization in order to obtain a persistent hydrophobic organic layer on the glass surface.

## Experimental Section

### Materials

All reagents were obtained from Sigma-Aldrich, except (chloromethyl)­trimethylsilane (Manchester Organics Limited). Organic solvents, drying agents (e.g., anhydrous magnesium sulfate), and inorganic chemicals (e.g., sodium bromide) were at least reagent grade and obtained from EUROCHEM BGD, CHEMPUR, P.P.H. “Stanlab” or Avantor Performance Materials. Tetrahydrofuran used for Grignard reactions was dried overnight with calcium hydride and distilled directly before use under protecting atmosphere (argon). Epoxy-terminated oligodimethylsiloxane (MCR-E11, MW = 1000 Da, viscosity 10–15 cSt) was obtained from Gelest, Inc., while amine-terminated oligodimethylsiloxane (L 656, kinematic viscosity 15–35 mm^2^/s) was purchased from Wacker (see Table 1 for their chemical structures and properties). Microscopic slide glasses (original dimensions: 76 mm × 26 mm) were obtained from ChemLand. Double-distilled water was used in all experiments.

**Table 1:**
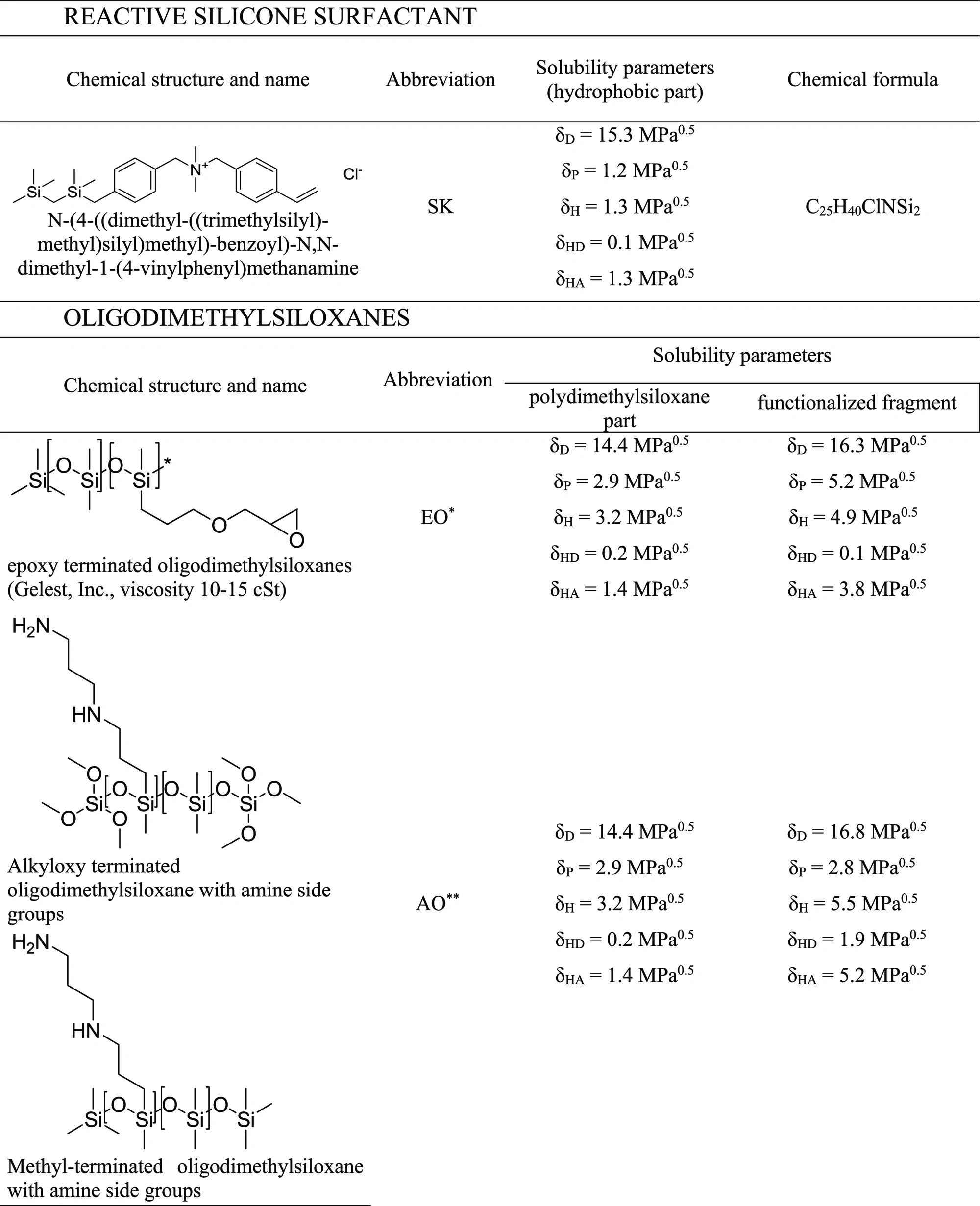
Structures and Properties of the Studied Reactive Silicone Surfactant and Oligodimethylsiloxanes

^*^ Values of Δδ for siloxane part: 3.12 MPa^0.5^ (eq 1) and 2.49 MPa^0.5^ (eq 2); values of Δδ for epoxy part: 5.74 MPa^0.5^ (eq 1) and 4.47 MPa^0.5^ (eq 2).

^**^ Values of Δδ for siloxane part: 3.12 MPa^0.5^ (eq 1) and 2.49 MPa^0.5^ (eq 2); values of Δδ for amine part: 5.40 MPa^0.5^ (eq 1) and 5.06 MPa^0.5^ (eq 2).

### Synthesis of Reactive Silicone Surfactant

Reactive silicone surfactant, *N*-(4-((dimethyl-((trimethylsilyl)-methyl)­silyl)­methyl)-benzoyl)-*N*,*N*-dimethyl-1-(4-vinylphenyl)­methanamine (SK), was obtained in a multistep route, comprising exchange of chloride motif in (chloromethyl)­trimethylsilane with bromide one (i), preparation of chlorodimethyl­((trimethylsililo)­methyl)­silane under Grignard conditions (ii), bromination of p-xylene to 1,4-bis­(bromomethyl)­benzene (iii), coupling of 1,4-bis­(bromomethyl)­benzene with chlorodimethyl­((trimethylsililo)­methyl)­silane under Grignard conditions to yield 4-(bromomethyl)­benzyldimethyl­((trimethylsilil)­methyl)­silane (iv), preparation of 1-(4-((dimethyl­((trimethylsilil)­methyl)­silil)­methyl)-phenyl)-*N*,*N*-dimethylmethanamine (v) and its quaternization with 1-(chloromethyl)-4-vinylbenzene to afford final reactive surfactant—*N*-(4-((dimethyl-((trimethylsilyl)-methyl)­silyl)­methyl)-benzoyl)-*N*,*N*-dimethyl-1-(4-vinylphenyl) methanamine (vi). The whole synthetic route is shown in Scheme 1.

**Scheme 1:**
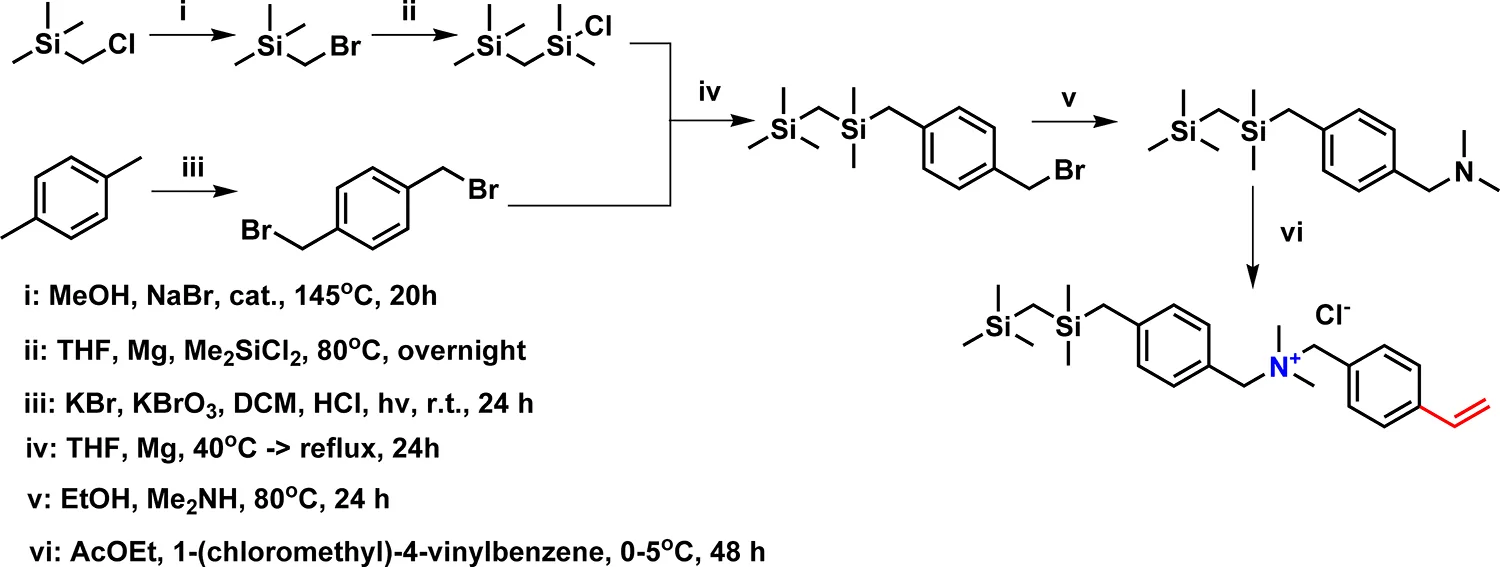
General Synthetic Route for Reactive Silicone Surfactant

The exchange of the chloride motif in (chloromethyl)­trimethylsilane with the bromide one (step (i)) was performed in methanol under an atmosphere of dry nitrogen. Briefly, (chloromethyl)­trimethylsilane (37.5 g, 0.31 mol), anhydrous sodium bromide (62.0 g, 0.60 mol), tetrabutylammonium bromide (7.53 g, 0.023 mol—the catalyst), and 200 mL of methanol were placed in a pressure vessel equipped with a heating mantle and mechanical stirrer. The reaction mixture was kept at 145 °C for 20 h under intensive stirring, followed by cooling to room temperature, mixing with 500 mL of distilled water, and liquid–liquid extraction (dichloromethane, 190 mL). The organic layer was dried over anhydrous MgSO_4_ and carefully filtered, while dichloromethane was distilled off under slightly reduced pressure (505 mbar at 40 °C). The crude product was fractionally distilled, yielding (bromomethyl)­trimethylsilane (38.78 g, 0.23 mol, yield: 75%). ^1^H NMR (CDCl_3_): δ (ppm) 0.08–0.10 ppm (Si­(C**H**_3_)_3_, 9H), 2.40 ppm (SiC**H**_2_Br).

The preparation of chlorodimethyl­((trimethylsililo)­methyl)­silane under Grignard conditions (step (ii)) was performed in dry tetrahydrofuran under an atmosphere of dry argon. For the first step, magnesium turnings (1.48 g, 0.06 mol) and a catalytic amount of iodine (ca. 10 mg) were placed in a three-neck bulb, filled with 30 mL of THF, and heated to 80 °C, followed by introduction of (bromomethyl)­trimethylsilane (12.27 g, 0.07 mol) via a dropping funnel. The reaction was continued at reflux until whole magnesium was reacted (around 2 h). For the second step, the green-colored reaction mixture was cooled to room temperature and placed in a dropping funnel, followed by its gradual introduction into the heated (80 °C) solution of dichlorodimethylsilane (30.97 g, 0.20 mol) in THF (30 mL). Heating at reflux was continued overnight, while the obtained mixture was cooled to room temperature, filtered, and THF was removed under reduced pressure. The crude chlorodimethyl­((trimethylsililo)­methyl)­silane was purified by vacuum distillation (bp 88.5 °C at 100 mmHg), giving 9.05 g (0.05 mol, yield 83%). ^1^H NMR (CDCl_3_): δ (ppm) 0.03–0.04 ppm (CH_2_Si­(C**H**_3_)_3_, 9H), 0.40–0.41 ppm (SiC**H**_2_Si­(C**H**_3_)_2_Cl).

The bromination of *p*-xylene to 1,4-bis­(bromomethyl)­benzene (step (iii)) was performed as a free-radical photoinitiated reaction. Potassium bromate (32.91 g, 0.2 mol), potassium bromide (120.71 g, 1 mol), AIBN (azobis­(isobutyronitrile)) -catalytic amount (ca. 20 mg), and *p*-xylene (21.23 g, 0.2 mol) were placed in a three-necked flask, equipped with a dropping funnel with concentrated hydrochloric acid (221 mL, 35–38%). The reaction mixture was irradiated with a UV lamp (wavelengths: 365 and 254 nm), and hydrochloric acid was slowly introduced into the mixture. After the whole amount of HCl was added, the reaction was continued (with irradiation) for 24 h. The reaction mixture was extracted with dichloromethane (3 × 30 mL), and the combined organic layers were dried over anhydrous MgSO_4_. After removal of dichloromethane under reduced pressure, the crude product was recrystallized from methanol, yielding 33.12 g (62.7%) of pure 1,4-bis­(bromomethyl)­benzene (experimental mp 145–148 °C).

The coupling of 1,4-bis­(bromomethyl)­benzene with chlorodimethyl­((trimethylsililo)­methyl)­silane to yield 4-(bomomethyl)­benzyldimethyl ((trimethylsilil)­methyl)­silane (step (iv)) was performed under Grignard conditions. Briefly, into a three-necked flask, magnesium turnings (0.97 g, 0.04 mol), a catalytic amount of iodine (around 10 mg), and THF (10 mL) were introduced, followed by the introduction of 1,4-bis­(bromomethyl)­benzene (15.84 g, 0.05 mol) in THF (50 mL) under gentle heating and an inert argon atmosphere. The reaction was continued at 40 °C until complete dissolution of magnesium (around 2 h), followed by gradual introduction of chlorodimethyl­((trimethylsililo)­methyl)­silane (8.05 g, 0.04 mol) via a dropping funnel and heating under reflux for 24 h. The reaction was quenched with 40 mL of saturated aqueous ammonium chloride, followed by introduction of diethyl ether (100 mL) and dichloromethane (100 mL) and extraction. The organic layer was washed with distilled water (2 × 50 mL), dried over anhydrous MgSO_4,_ and evaporated under reduced pressure. 4-(Bomomethyl)­benzyldimethyl­((trimethylsilil)­methyl)­silane was obtained as a semi-solid mass (5.44 g, 0.017 mol; yield: 42.5%). ^1^H NMR (CDCl_3_): δ (ppm) 0.03–0.04 ppm (Si­(C**H**_3_)_2_C**H**_2_Si­(C**H**_3_)_3_, 17H), 1.93–1.96 ppm (SiC**H**_2_Ar, 2H), 4.45 ppm (ArC**H**_2_Br, 2H), 7.07–7.35 ppm (C**H**(Ar), 4H).

The preparation of 1-(4-((dimethyl­((trimethylsilil)­methyl)­silil)­methyl)-phenyl)-*N*,*N*-dimethylmethanamine (step (v)) was performed in a closed vessel reactor. 4-(Bomomethyl)­benzyldimethyl­((trimethylsilil)­methyl)­silane (5.44 g, 0.017 mol) was mixed with excess of dimethylamine (45 mL, 33% solution in absolute ethanol) and placed in a closed vessel, followed by heating at 80 °C for 12 h. After cooling to room temperature, the reaction mixture was evaporated to dryness, and the residue was dissolved in water (50 mL), followed by extraction with ethyl acetate (50 mL). The organic layer was washed with water (2 × 25 mL), dried with anhydrous MgSO_4_, and evaporated to dryness under reduced pressure, yielding 1.58 g (0.0054 mol, yield 31.7%) of 1-(4-((dimethyl­((trimethylsilil)­methyl)­silil)­methyl)-phenyl)-*N*,*N*-dimethylmethanamine. ^1^H NMR (CDCl_3_): δ (ppm) −0.14 ppm (SiC**H**_2_Si, 2H), 0.04–0.10 ppm (Si­(C**H**_3_)_2_CH_2_Si­(C**H**_3_)_3_, 15H), 2.22 ppm (SiC**H**_2_Ar, 2H), 2.85–2.86 ppm (N­(C**H**_3_)_2_, 6H), 3.39 ppm (ArC**H**_2_N, 2H), 7.07–7.24 ppm (C**H**(Ar), 4H).

The quaternization of 1-(4-((dimethyl­((trimethylsilil)­methyl)­silil)­methyl)-phenyl)-*N*,*N*-dimethylmethanamine with 1-(chloromethyl)-4-vinylbenzene (step vi) was performed under mild conditions to avoid accidental polymerization of reagents and/or the final product. 1-(4-((Dimethyl­((trimethylsilil)­methyl)­silil)­methyl)-phenyl)-*N*,*N*-dimethylmethanamine (1.58 g, 0.0054 mol) and 1-(chloromethyl)-4-vinylbenzene (1.04 g, 0.006 mol) were dissolved in 20 mL of ethyl acetate and kept in a closed vessel at 0–5 °C for 48 h. The precipitated solid product—*N*-(4-((dimethyl-((trimethylsilyl)-methyl)­silyl)­methyl)-benzoyl)-*N*,*N*-dimethyl-1-(4-vinylphenyl) methanamine—was washed with cold ethyl acetate and dried overnight at room temperature under reduced pressure. Yield: 1.97 g (89%). ^1^H NMR (CDCl_3_): δ (ppm) 0.01–0.02 ppm (Si­(C**H**_3_)_2_C**H**_2_Si­(C**H**_3_)_3_, 17H), 2.02 ppm (SiC**H**_2_Ar, 2H), 3.33 ppm (N^+^(C**H**_3_)_2_, 6H), 4.72–4.74 ppm (ArC**H**_2_N^+^C**H**_2_Ar, 4H), 5.37–5.39, 5.61 ppm (=C**H**_2_, 2H), 6.61–6.65 ppm (=C**H**Ar, 1H), 7.07–7.61 ppm (C**H**(Ar), 8H).

### Characterization of Silicone Surfactant

^1^H NMR spectra were recorded on a Bruker AMX-500 instrument for samples dissolved in CDCl_3_ at concentrations of 5–10 mg/mL. Chemical shifts are given in ppm and related to the residual peak of CHCl_3_ (7.26 ppm) as an internal standard. The data processing was performed utilizing Bruker TopSpin (version 3.6.1). FT-IR spectra were obtained in ATR mode using a Nicolet iS10 FT-IR spectrometer (Thermo Scientific, Waltham, MA, USA), covering the range of 600–4000 cm^–1^. Each spectrum consisted of 32 scans and was recorded at a resolution of 4 cm^–1^.

### Glass Hydrophobization

Glass hydrophobization was performed utilizing three approaches: spray coating, spin coating, and dip coating. Glass plates (2.6 cm × 2.6 cm) were cut from microscope slide glasses and purified by procedures for glass substrates according to Ossila (washing with 2% Hellmanex III solution, distilled water, and isopropanol, followed by UV/ozone treatment), similarly as described by Bykkam et al.^16^ All samples, directly after the layer preparation, were irradiated with a UV lamp (wavelengths: 365 and 254 nm) for 20 min in order to execute reactive surfactant polymerization and/or Michael addition (when amine-terminated oligodimethylsiloxane was used). Compositions and conditions for sample preparation (all were solutions in isopropanol) are shown in Table 2 together with values of contact angles and surface energies. Spray coating was performed using an air-driven atomizer, fed with appropriate organic solutions, for three different distances between the head and the glass: far (60 cm), medium (30 cm), and proximate (15 cm). Casting of the layer by spin coating was performed using an Ossila standard spin coater with a custom-made, 3D-printed chuck for three different rotational speeds: 1000, 1500, and 2000 rev/min, with spinning time equal to 20 s. The glasses were completely covered with appropriate solutions prior to the spinning sequence. Dip coating was performed using an Ossila Dip Coater with a 3D-printed chamber for solutions (dimensions: 31.6 mm × 4.6 mm × 30.2 mm) for three different withdrawal speeds: 0.1, 0.2, and 0.4 mm/s, and the procedure was repeated once, twice, or four times (see Table 2).

**Table 2:** Values of Contact Angles for Water (θ_H_2_O_) and Diiodo-methane (θ_DIM_) and Surface Energies (γ_s_) (Disperse (γ_s_^d^) and Polar (γ_s_^p^) Components) for the Particular Samples

		spray-coating samples				
samples abbreviation^a^ description		γ_s_ [mJ/m^2^]	γ_s_^d^ [mJ/m^2^]	γ_s_^p^ [mJ/m^2^]	θ_H_2_O_ [°]	θ_DIM_ [°]
1E_d1	1% EO, d1	52.9 ±1.4	25.9 ±0.2	27.0 ±1.1	47.8 ±1.6	64.7 ±0.4
1E_d2	1% EO, d2	54.7 ±0.8	30.4 ±0.4	24.4 ±0.4	47.5 ±0.4	56.9 ±0.8
1E_d3	1% EO, d3	57.6 ±0.7	25.4 ±0.2	32.2 ±0.5	40.5 ±0.6	65.6 ±0.4
1E_0.1S_d1	1% EO, 0.1% SK, d1	52.1 ±0.5	25.0 ±0.2	27.0 ±0.3	48.6 ±0.4	66.2 ±0.3
1E_0.1S_d2	1% EO, 0.1% SK, d2	-	-	-	-	-
1E_0.1S_d3	1% EO, 0.1% SK, d3	46.3 ±0.7	32.6 ±0.2	13.6 ±0.4	62.8 ±0.7	52.9 ±0.4
0.5E_0.5S_d1	0.5% EO, 0.5% SK, d1	51.2 ±0.8	34.4 ±0.2	16.9 ±0.5	56.2 ±0.8	49.8 ±0.4
0.5E_0.5S_d2	0.5% EO, 0.5% SK, d2	53.3 ±1.3	23.7 ±0.3	29.6 ±1.0	46.3 ±1.3	68.5 ±0.5
0.5E_0.5S_d3	0.5% EO, 0.5% SK, d3	65.6 ±0.4	35.6 ±0.2	29.9 ±0.2	33.0 ±0.4	47.5 ±0.3
1A_0.5S_d1	1% AO, 0.5% SK, d1	25.4 ±0.4	20.2 ±0.2	5.2 ±0.2	90.6 ±0.5	74.9 ±0.4
1A_0.5S_d2	1% AO, 0.5% SK, d2	30.2 ±1.2	22.0 ±0.3	8.2 ±0.9	80.6 ±1.7	71.5 ±0.5
1A_0.5S_d3	1% AO, 0.5% SK, d3	35.9 ±0.9	21.6 ±0.3	14.3 ±0.6	70.2 ±0.8	72.3 ±0.6

		spin-coating samples				
samples abbreviation^b^ description		γ_s_ [mJ/m^2^]	γ_s_^d^ [mJ/m^2^]	γ_s_^p^ [mJ/m^2^]	θ_H_2_O_ [°]	θ_DIM_ [°]
1E_r1	1% EO,r1	36.4 ±2.5	31.2 ±0.8	5.2 ±1.7	80.7 ±4.0	55.4 ±1.4
1E_r2	1% EO, r2	42.4 ±0.8	32.3 ±0.4	10.1 ±0.4	69.5 ±0.7	53.5 ±0.8
1E_r3	1% EO, r3	39.0 ±0.7	31.6 ±0.4	7.4 ±0.4	75.5 ±0.7	54.8 ±0.7
1E_0.1S_r1	1% EO, 0.1% SK, r1	56.0 ±0.5	33.2 ±0.2	22.8 ±0.3	47.5 ±0.4	52.0 ±0.4
1E_0.1S_r2	1% EO, 0.1% SK, r2	59.8 ±0.6	32.8 ±0.3	27.0 ±0.3	41.1 ±0.5	52.6 ±0.5
1E_0.1S_r3	1% EO, 0.1% SK, r3	56.0 ±0.4	32.6 ±0.1	23.4 ±0.3	47.0 ±0.4	53.0 ±0.2
0.5E_0.5S_r1	0.5% EO, 0.5% SK, r1	71.2 ±0.5	34.8 ±0.3	36.4 ±0.2	19.9 ±0.4	49.0 ±0.5
0.5E_0.5S_r2	0.5% EO, 0.5% SK, r2	72.1 ±0.7	37.0 ±0.4	35.1 ±0.3	19.5 ±0.5	45.1 ±0.8
0.5E_0.5S_r3	0.5% EO, 0.5% SK, r3	70.5 ±0.4	35.4 ±0.2	35.0 ±0.2	17.3 ±0.2	47.9 ±0.5
1A_0.5S_r1	1% AO, 0.5% SK, r1	33.6 ±1.9	26.2 ±0.8	7.4 ±1.1	79.4 ±2.1	64.1 ±1.4
1A_0.5S_r2	1% AO, 0.5% SK, r2	26.2 ±1.0	24.3 ±0.5	1.9 ±0.5	95.8 ±2.3	67.5 ±0.9
1A_0.5S_r3	1% AO, 0.5% SK, r3	26.7 ±2.2	22.7 ±1.2	4.0 ±1.0	90.4 ±2.4	70.3 ±2.2

		dip-coating samples				
samples abbreviation^c^ description		γ_s_ [mJ/m^2^]	γ_s_^d^ [mJ/m^2^]	γ_s_^p^ [mJ/m^2^]	θ_H_2_O_ [°]	θ_DIM_ [°]
1A_0.5S_1w1	1% AO, 0.5% SK, 1w1	27.3 ±0.4	23.7 ±0.3	3.6 ±0.1	90.2 ±0.3	68.5 ±0.3
1A_0.5S_1w1’	1% AO, 0.5% SK, 1w1’	37.3 ±0.4	30.9 ±0.2	6.4 ±0.2	78.0 ±0.5	55.9 ±0.4
1A_0.5S_1w2	1% AO, 0.5% SK, 1w2	27.4 ±0.5	22.2 ±0.3	5.2 ±0.2	87.3 ±0.4	71.2 ±0.6
1A_0.5S_1w2’	1% AO, 0.5% SK, 1w2‘	30.3 ±0.3	24.7 ±0.1	5.6 ±0.1	84.2 ±0.3	66.8 ±0.2
1A_0.5S_1w3	1% AO, 0.5% SK, 1w3	29.3 ±0.4	23.5 ±0.2	5.8 ±0.3	85.2 ±0.6	68.9 ±0.3
1A_0.5S_2w1	1% AO, 0.5% SK, 2w1	31.4 ±0.5	24.8 ±0.3	6.5 ±0.2	82.1 ±0.5	66.5 ±0.5
1A_0.5S_2w2	1% AO, 0.5% SK, 2w2	28.1 ±0.4	25.8 ±0.1	2.3 ±0.3	93.2 ±0.7	64.9 ±0.2
1A_0.5S_2w3	1% AO, 0.5% SK, 2w3	35.0 ±0.5	27.6 ±0.3	7.4 ±0.3	78.0 ±0.3	61.7 ±0.5
1A_0.5S_4w1	1% AO, 0.5% SK, 4w1	33.8 ±0.7	28.5 ±0.3	5.3 ±0.4	82.3 ±1.1	60.1 ±0.5
1A_0.5S_4w2	1% AO, 0.5% SK, 4w2	35.1 ±0.3	28.4 ±0.1	6.7 ±0.2	79.0 ±0.5	60.3 ±0.2
1A_0.5S_4w3	1% AO, 0.5% SK, 4w3	34.4 ±0.5	30.3 ±0.1	4.1 ±0.4	84.1 ±1.1	56.9 ±0.2

a Distance between the head and the glass surface: 60, 30, or 15 cm—samples d1, d2, and d3, respectively.

b Rotation speed: 1000, 1500, or 2000 rpm—samples r1, r2, and r3, respectively; spinning time: 20 s.

c Withdrawal speed: 0.1, 0.2, or 0.4 mm/s—samples w1, w2, and w3, respectively; number of dip cycles: 1, 2, 4—the right number before w1, w2, w3; marker (‘): glass side not exposed to UV.

Moreover, in order to study the pure hydrophobic layer by XPS, the spin-coating procedure was performed for the optimized system using glass slides with the sputtered gold layer. Gold sputtering was performed using a Quorum magnetron sputter with a sputtering time equal to 300 s and a current of 20 mA.

Additionally, the durability of the deposited coatings to water and isopropyl alcohol was determined. For this purpose, after six months of storage at room temperature, tests were conducted involving 1 min contact of the layers with distilled water and three temporary immersions in isopropanol. After thoroughly drying the samples in an inert gas stream, their contact angles were measured according to the procedure described in section Surface Energy Determination. The tests were conducted for selected samples obtained by spray, spin, and dip coating.

### HSPiP Calculations

The mutual solubility/compatibility between the new reactive surfactant (SK) and both oligodimethylsiloxane derivatives (epoxy–EO- and amine–AO-terminated) were calculated utilizing the Y-MB approach by HSPiP software. The structures of particular components (poly­(dimethylsiloxane), siloxane with epoxy side motif, siloxane with amine side motifs, and hydrophobic fragment of reactive silicone surfactant) were provided as SMILES notation. Due to the lack of appropriate group increments for ionic motifs, there was a possibility to perform calculations only for the hydrophobic fragment of SK. Solubility parameter components of dispersion (δ_D_), polar (δ_P_), and hydrogen bonding (δ_H_) were calculated, as well as the splitting of δ_H_ into donor (δ_HD_)/acceptor (δ_HA_) counterparts. The distance (Δδ) between appropriate substances/counterparts (subscripts 1 and 2) was calculated according to equations:

1 $${\left(\Delta \delta \right)}^{2}=4{\left(\delta_{D2}-\delta_{D1}\right)}^{2}+{\left(\delta_{P2}-\delta_{P1}\right)}^{2}+{\left(\delta_{H2}-\delta_{H1}\right)}^{2}$$

2 $${\left(\Delta \delta \right)}^{2}=4{{(\delta }_{D2}-\delta_{D1})}^{2}+{{(\delta }_{P2}-\delta_{P1})}^{2}+2\left(\delta_{HD2}-\delta_{HD1}\right)\left(\delta_{\mathrm{HA}2}-\delta_{\mathrm{HA}1}\right)$$

for total (eq 1) and split (eq 2) values of δ_H_, respectively.

### Surface Energy Determination

One of the most commonly used methods for determining surface free energy (γ_s_) is the Owens–Wendt–Rabel–Kaelble model (OWRK), which assumes that this energy is the sum of disperse (γ_s_^d^) and polar (γ_s_^p^) interaction components^19,20,32,33,19,20,32,33^

3 $$\gamma_{s}=\gamma_{s}^{d}+\gamma_{s}^{p}$$

In this method, to determine both contributions, it is necessary to use two liquids with known values of γ_l_, γ_l_^d^, and γ_l_^p^ and solve the appropriate system of equations using the following relationship.

4 $$\gamma_{l}\left(1+\cos\theta \right)=2\sqrt{\gamma_{l}^{d}\gamma_{s}^{d}}+2\sqrt{\gamma_{l}^{p}\gamma_{s}^{p}}$$

where θ represents the contact angle between the droplet and the coating surface; γ_s_ and γ_l_, represent the surface energy of the coating and the surface tension of the liquid, respectively. γ_s_^d^ and γ_s_^p^ represent the disperse and polar fractions of the surface, respectively. γ_l_^d^ and γ_l_^p^ are the disperse and polar fractions of the liquid, respectively. The γ_l_, γ_l_^d^, and γ_l_^p^ values are 72.8, 21.8, and 51.0 mJ/m^2^ for water and 50.8, 50.8, and 0.0 mJ/m^2^ for diiodo-methane, respectively.^33^

Contact angles for water (θ_H_2_O_) and diiodo-methane (θ_DIM_) were measured with a goniometer DSA25, Krüss GmbH, Germany, at room temperature (23 °C). The double sessile drop method with a droplet volume of 3 μL was used. Measurements were performed for each coating by measuring 4 different positions on the same sample; the results were then averaged, and the standard deviation was calculated.

### Atomic Force Microscopy and Surface Roughness Determination

Atomic force microscopy (AFM) imaging was performed on identically prepared specimens, with additional topographic measurements acquired on uncoated substrate surfaces for reference. All AFM measurements were executed in intermittent contact (tapping) mode on a Nanosurf NaioAFM instrument utilizing a pyramidal tip shape NANOSENSORS PPP-NCLAuD cantilever with a resonance frequency of 190 kHz and a force constant of 48 N/m. Each image was acquired using the same scan rate of 1 μm/sek. A standard image consisted of 512 scan lines, with 512 data points recorded per line, resulting in a resolution of 512 × 512 pixels. The samples were scanned over the AFM operating range, using scan sizes of 50, 25, 10, and 5 μm. Postacquisition image processing and surface roughness analysis were carried out using WSxM software.^34^ Images of the same size and resolution were used for comparison and RMS determination.

### Raman Microscopy Measurements

Raman microscopy measurements were performed in the Laboratory of Scanning Electron Microscopy, Institute of Geological Sciences, University of Wrocław, using a Renishaw inVia Qontor apparatus, equipped with a Renishaw Centrus detector (resolution 1040 px × 256 px), 532 nm laser, and diffraction grating (1800 lines per millimeter). The equipment was calibrated with a silicon standard (520.5 cm^–1^). All mapping measurements (for samples 1A_0.5S_r2, 1A_0.5S_1w1, and 1A_0.5S_2w2 in Table 2) were executed with a laser power equal to 13.5 mW (signal acquisition time: 3 s) in the range of 1517.4–3016.7 cm^–1^ with a slit opening of 65 μm and a center of 2378 μm. Single-point measurements for glass and reactive silicone surfactant were executed in ranges of 199.1–3198.1 cm^–1^ and 400.5–3499.2 cm^–1^, respectively. All measurements were assisted by an optical microscope (magnification x50).

We used Raman spectra to extract information on the position-dependent distribution of materials. However, the raw spectra contain a large number of redundant information. To enable an effective comparison, we refined the Raman data by extracting the most relevant features using a principal component analysis (PCA) model. In this approach, each spectrum measured at a given (*x*, *y*, *z*) point (originally consisting of 1015 data points) is compressed into 10 principal components. To quantify the similarity between such reduced spectra (and thus identify regions corresponding to the same material), we computed the distance in the resulting 10-dimensional PCA space. The processed data then served as the input for a subsequent clustering procedure within the K-means algorithm. The PCA and K-means calculations were done using the scikit-learn library^35^ in Python.

### XPS Studies with Ar^+^ Sputtering

X-ray photoelectron spectroscopy (XPS) was employed to evaluate the surface chemistry of the prepared layers. All spectroscopic measurements were conducted using a nonmonochromatized Mg Kα X-ray source (1253.6 eV) on an EA10 AES/XPS instrument (Leybold-Heraeus GmbH, Cologne, Germany). The experiments were performed under ultrahigh vacuum conditions (chamber pressure <5 × 10^–8^ mbar) at ambient temperature. The spectrometer energy scale was calibrated against the Au4*f*, Ag3*d*, and Cu2*p* standard lines, while all binding energies were charge-corrected to the adventitious C1s peak at 285.0 eV. The instrumental spectral resolution was assessed as 0.96 eV from the FWHM of the Ag3*d*_5/2_ reference line. Data acquisition was managed using WSpectra v8 (R. Unwin, 2001), and subsequent spectral processing, including peak deconvolution, was executed in CasaXPS v2.3 (Casa Software Ltd., Teignmouth, UK).^36^ An initial survey scan was recorded to identify surface composition, after which high-resolution regional scans were acquired for the relevant elemental photoelectron lines. Quantitative atomic percentages were calculated from the integrated peak areas, corrected for the respective sensitivity factors (RSF) of each detected element.

Depth profiling was achieved through sequential Ar^+^ ion sputtering utilizing a PREVAC IS 40C1 ion gun. The purity of Ar gas (Linde, Argon 5.0, HiQ MINICAN) used in the etching process was 5N. The etching protocol was executed with a current density of 2 μA/cm^2^, an accelerating potential of 1 kV, an argon partial pressure of 5 × 10^–5^ mbar, and a working distance of 30 mm from the sample surface. The depth profiling measurement procedure was based on the cyclic repetition of XPS measurements followed by Ar^+^ surface etching. This sputtering sequence was continued until the emergence of core-level spectral features indicative of the bulk glass material. To determine the etching rate of the deposited layer during the etching process, additional calibration measurements were performed for the layer deposited on the gold buffer layer. This allowed us to determine the etching rate of the layer by cyclic etching until a signal from gold (which is not present in the deposited layer) appeared. This approach does not require determining the ion etching efficiency; it experimentally determines the etching rate based on a reference, which in this case is the gold buffer layer. Gold was chosen as the buffer layer due to the good separation of XPS emission peaks (Au4*f*_7/2_ at 84 eV vs pure Si2*p* at around 99 eV or Si2p at 103 eV in SiO_2_) and the neutrality of gold toward both the substrate and the adsorbate. This procedure requires only one additional calibration sample with the buffer layer to perform calibration measurements. Such a procedure corresponds to the classical estimation of the ion etching rate using a standard such as SiO_2_/Si.^37–39^

For XPS characterization, specimens were fabricated via two distinct deposition methodologies: dip coating and spin coating. Samples obtained by spray coating were too rough (see results of AFM studies) for Raman microscopy and XPS spectroscopy analyses. Spectroscopic analyses were conducted on both as-deposited (pristine) samples and surfaces subjected to postdeposition ion etching.

## Results and Discussion

### Design, Synthesis, and Structure Determination of Reactive Silicone Surfactants

The design of our novel carbosilane reactive surfactants takes into consideration the following aspects, crucial for their performance: (i) thermal and chemical stability; (ii) rigidity of the hydrophobic fragment; (iii) compatibility with commercial, reactive oligosiloxanes; and (iv) the choice of an appropriate, polymerizable motif, including its location within the molecule (inside hydrophilic or hydrophobic parts, within the counterion or linking motif). Short-chain siloxane surfactants, especially of ionic origin, are characterized by a flexible but hydrolysis-susceptible hydrophobic unit.^3^ Conversely, carbosilane surfactants, far less studied in the literature, possess a more rigid but chemically stable hydrophobic motif.^3^ Typically, reactive silicone surfactants possess so-called cleavable motifs, e.g., ester ones, between hydrophobic and hydrophilic counterparts.^7,9^ One way to obtain stable and easy-to-apply hydrophobic coatings is to use reactive silicone surfactants capable of anchoring to the surface and then polymerizing to form a thin, insoluble layer. The polymerizable moiety may include, among others, an epoxy ring or a double bond. Due to the possibility of polymerization using radical or ionic mechanisms, the double bond, e.g., the vinyl moiety, is of greatest importance. Its reactivity depends primarily on the chemical nature of the attached substituent, including electronic (resonance and induction) and steric effects. Poly­(dimethylsiloxanes) (PDMS) bearing vinyl ether end-groups were tested as a silicone wetting agent for the UV-cured coatings.^40^ Electron-donating groups (e.g., alkyl, ether groups) increase the electron density of the C=C bond, which hinders free-radical polymerization. In the case of substituents containing conjugated systems (e.g., aromatic rings), delocalization of π-electrons stabilizes the transition radical, significantly increasing the susceptibility to free-radical polymerization. For example, the introduction of vinyl-terminated siloxanes has been studied in terms of the impact on aggregation in aqueous systems.^10^ However, the direct reactivity of vinyl and allyl siloxanes is limited, and harsh conditions may need to be considered due to the decreasing reactivity of oligomeric molecules with chain length,^41^ in contrast to styrene or methacrylate derivatives, known for their high tendency to polymerize upon initiation in mild conditions. The use of mixed reactive surfactants (Noigen RN10, vinyl-modified ethoxylated alkylphenol) and a siloxane derivative for surface modification has been demonstrated for clay particle stabilization.^42^ On the other hand, the use of hydrocarbon-based surfactants limits the maximal level of hydrophobization due to minimal surface tension, when compared with silicone-based amphiphiles.^1,3^ The location of the polymerizable motif within the hydrophilic part, especially when directly attached to an aromatic ring, seems to be beneficial due to optimal reactivity toward both the hydrophilic surface and the hydrophobic siloxane oligomer matrix.^42^ Moreover, it should be emphasized that some structural similarities, e.g., the presence of aromatic rings, between both hydrophilic and hydrophobic parts of the surfactant molecule may enhance its compatibility and reactivity with polar and nonpolar surfaces and environments. After considering all of the aforementioned aspects, as well as the possible synthetic routes, we have designed a carbosilane cationic surfactant, comprising both aliphatic (methyl or methylene) and aromatic motifs within the hydrophobic fragment, as well as a vinyl polymerizable group attached to an aromatic ring in the hydrophilic moiety.

The synthesis of reactive silicone surfactant was performed using routes and methodologies devised by ourselves, including chloride-to-bromide exchange (step (i)), two couplings under Grignard conditions (steps (ii) and (iv)), free-radical bromination (step (iii)), introduction of a tertiary amine motif by alkylation (step (v)), and final quaternization (step (vi)) (see Scheme ). Our novel surfactant—according to the best of our knowledge—constitutes a completely new class of surface-active compounds—reactive cationic silicone surfactants, comprising exclusively Si–C and C–C bonds in both hydrophobic and hydrophilic counterparts, with the exception of a C–N bond for the quaternary ammonium headgroup. It should be emphasized that the surfactant design meets requirements for so-called custom-designed products, i.e., compounds synthesized to fulfill specific practical requirements.

The crucial final step, comprising introduction of hydrophilic (quaternary ammonium chloride) and reactive (polymerizable vinyl) motifs, is performed under very mild conditions (reduced temperature, organic solvent of neutral character), enabling amphiphilic product precipitation directly from the reaction mixture. It should be noted that the obtained product was dried under reduced pressure and kept in a dark, dry, and cool place in order to avoid its premature degradation or polymerization.

### Glass Surface Hydrophobization by Spray-, Spin-, and Dip-Coating Approaches

The deposition of a thin organic layer on a flat surface, e.g., glass, may be performed utilizing numerous approaches, including the most popular and industrially acceptable ones: spray, spin, and dip coating. The main differences between them are connected with their tolerance to surface shape (in general, spray coating may be applied not only to flat surfaces but also to more complicated devices, etc.) and film casting kinetics (when a volatile solvent is used, spray and spin coating typically involve their rapid evaporation, while dip coating, especially for slow withdrawal, comprises a rather gradual process limited to the three-phase contact point). Therefore, the layer features may significantly differ according to the casting method.

Another crucial parameter for all of the studied methodologies is the composition of the hydrophobic, organic layer. Since the carbosilane surfactant is characterized by rigidity of the hydrophobic moiety,^3^ the second ingredient—functional silicone oligomer—needs to be characterized by excellent flexibility in order to assume the layer compatibility with glass surface roughness. Oligodimethylsiloxanes with amine function are known for great flexibility, easy accommodation to the surface, and reversible reactivity, so they may be used as components for self-healing elastomers^43–47^ and fiber-borne coatings.^48^ Moreover, amine-terminated oligodimethylsiloxane (L 656, Wacker) comprises a mixture of alkyloxy-terminated and methyl-terminated oligodimethylsiloxane with amine side groups, so it may bind to the glass surface via reversible (methyl-terminated component) and irreversible (alkyloxy-terminated derivative) processes.

Oligodimethylsiloxanes with epoxy-reactive motifs may be used for 3D printing materials,^49^ functionalized inorganic–organic hybrid materials,^50^ and as complex monomer precursors.^51^ It should be noted that epoxide-functionalized siloxanes may provide the synthesis of elastic structures like elastomers^50^ and thus have potential for accommodation of more rigid compounds. Moreover, taking into account our aim for the easy formation of a polymeric structure on the glass surface, the epoxy motif in the siloxane derivative may undergo photoinitiated polymerization.^51^ The main obstacle in the field of surface hydrophobization is the possibility of an epoxide ring-opening reaction yielding a hydrophilic hydroxide motif, especially on the external layer.

In order to study the compatibility of the obtained surfactant—in fact, its hydrophobic part—with particular counterparts (poly­(dimethylsiloxane) and functional siloxane) of commercially available silicones (see Table 1), we have calculated the Hansen solubility parameter distance (Δδ) according to equations for total (see eq 1) and split (see eq 2) values of δ_H_. The use of both approaches is motivated by the character of the used commercial compounds, comprising oligomeric structures with a few or a few dozen particular building blocks (mers). In general, for simple, i.e., small molecular weight products, the first equation, considering the total value of δ_H_, is preferred, while for polymers, the second one seems to be more accurate. The compatibility between the oligomer and the hydrophobic part of the reactive surfactant is crucial for the stability of the layer, since the hydrophilic part of the surfactant is particularly responsible for attaching to the glass surface. It should be noted that the obtained Δδ values cannot be explicitly accounted for as a compatibility metric for the whole surfactant molecule, since calculations were performed exclusively for the hydrophobic fragment. According to both equations, the hydrophobic part of the reactive surfactant is compatible with poly­(dimethylsiloxane) (values of Δδ < 5 MPa^0.5^). On the other hand, its compatibility with epoxy- or amine-terminated siloxanes is considered to be limited (values of Δδ around 5 MPa^0.5^), indicating the possibility of metastable mixture formations. Therefore, the deposition method (spray, spin, or dip coating) may have a crucial impact on the structure and stability of the hydrophobic layer.

The assessment of the degree of hydrophobization using coatings based on oligodimethylsiloxanes and a synthesized reactive carbosilane surfactant (presented in Table 1) was performed using surface free energy (γ_s_) measurements based on the water and diiodo-methane contact angles. OWRK method is often used when testing siloxanes (and silanes) because these materials are known for their strong hydrophobic properties and low γ_s_. Using the OWRK method allows for precise assessment of how chemical modifications (e.g., amination) affect the polar and dispersion components of these coatings. In this way, the surface free energy is divided into a polar part (γ_s_^p^) and a dispersive part (γ_s_^d^). Results are presented in Table 2. Pure siloxanes (e.g., poly­(dimethylsiloxane)—PDMS), because their chains are shielded by nonpolar methyl groups, exhibit very low total surface energy (often in the range of 20–25 mJ/m^2^). Their polar component is close to zero, and the energy is almost exclusively due to dispersive interactions.^32^ The introduction of an epoxy or amine group to oligosiloxane chains enables the formation of dipole interactions and hydrogen bonds with glass surface functional groups, thereby increasing the coating’s adhesion. This also results in an increase in the surface free energy, resulting primarily from an increase in the polar component to several mJ/m^2^. This effect was observed for coatings formed with epoxy-terminated oligodimethylsiloxane, in which the polar components in the spin-coating method ranged from 5 to 10 mJ/m^2^. Based on the polar component of the surface energy for samples 1E_d1–3 (24–32 mJ/m^2^), we can also conclude that the glass surface was not uniformly covered by the spray-coating method. Moreover, the addition of SK reactive surfactant to coatings based on epoxy-terminated oligodimethylsiloxanes only slightly reduced the polar component of surface energy in spray-coated coatings. Whereas, in the case of spin coating, we observed significant increases in the polar component after adding a surfactant. Probably, too rapid solvent evaporation in the formed thin film likely caused uneven cross-linking, which limited the formation of appropriate siloxane chain conformations and resulted in a heterogeneous coating. The polar component values for these samples (1E_0.1S_r1–3 and 0.5E_0.5S_r1–3) increased three- to four-fold.

Significantly better-quality coatings (in both spin- and spray-coating methods) were produced from compositions containing methyl/alkyloxy-terminated oligodimethylsiloxane with amine side groups, for which the polar components ranged from 5 to 14 mJ/m^2^ for spray coating and from 2 to 7 mJ/m^2^ for spin coating. Since amino groups can form stronger hydrogen bonds with hydroxyl groups on the glass surface and their side, rather than terminal, arrangement in oligosilane chains provides more favorable conformations of polymer chains during coating, only composition 1A_0.5S, containing 1% aminooligosiloxane and 0.5% of the tested reactive surfactant, was selected for the evaluation of coating formation by the dip method. The results indicate that neither the withdrawing speed nor the number of dip cycles affected the degree of surface hydrophobization. We observed a nearly 2-fold reduction in the total surface energy compared with glass, with the polar component reduced 4-fold (2–7 mJ/m^2^), resulting in a 2-fold reduction in water wettability. The average contact angle for a clean glass plate is 54.2°. It is clear that the average contact angle values for modified glass plates are higher (θ_H_2_O_ = 78°–96°), but these coatings are still classified as hydrophilic (the contact angle for a hydrophilic surface is in the range of 0°–90°^52^) except for a few AO-based samples for which the contact angles were higher than 90° (see Table 2). The obtained results are similar to the literature values for coatings obtained from reactive siloxanes. The contact angles for coatings prepared using acryloxy-functionalized linear polysiloxanes are in the range of 73°–107°.^53^ The ability of alkyl- and fluoroalkyltriethoxysilanes to modify the wettability of glass was achieved at a similar level (θ_H_2_O_ = 82°–97°).^54^ It should be noted that, due to environmental reasons, the use of amphiphilic fluorine derivatives should be limited when there exist suitable alternatives.

The coatings’ durability was assessed by conducting wetting angle tests for selected samples obtained by spray (1A_0.5S_d1), spin (1A_0.5S_r2), and dip (1A_0.5S_1w2) coating, after six months, by exposing them to water and isopropanol (in accordance with the procedure described in section Glass Hydrophobization). Each step was assisted by surface energy determination, the data are gathered in Table S1 in ESI, together with the data for pure glass substrates. In general, for all of the studied samples, total surface energies slightly (by less than 5 mJ/m^2^) dropped after storage for six months, most possibly due to evaporation of solvent traces and the layer ripening, i.e., completion of all chemical processes within the polymer/oligomer matrix and at the boundary with glass. Contact with water, followed by careful drying in an inert gas stream, did not significantly change contact angles (for both water and diiodo-methane) and surface energies for samples obtained by spin and dip coating. Conversely, washing with isopropanol left the layer obtained by spin coating practically intact, while significantly changed the dip-coating layer, particularly in terms of water contact angle (dropping by around 10°, changing its properties from hydrophobic to hydrophilic). Such behavior additionally proves the durability and stability of the layer obtained by spin coating. The particular measurements before and after treatment with water and isopropanol for the spray-coating layer showed significant changes, balancing between the layer hydrophobicity and hydrophilicity, which corresponds with the sample’s high root-mean-square (RMS) values.

The results of our studies are consistent with Hansen solubility parameter calculations—in general, fast processes (especially spin coating) enable sufficient solubility of the reactive surfactant in the oligomer matrix, followed by their reaction, while relatively slow ones (dip coating) allow separation and deposition of SK. The deposits of the reactive surfactant, especially if present in crystalline or semicrystalline form, are not prone to undergo polymerization due to spatial constraints.

### Characterization of Hydrophobic Layer by AFM, Raman Microscopy, and XPS Spectroscopy

The characterization of the surface layer, responsible for glass hydrophobization, should include appropriate microscopic and spectroscopic techniques. The most important points of interest should include: (i) the layer homogeneity, (ii) the presence of any specific “interlayer” between the glass and the actual hydrophobic layer, (iii) the possibility of glass roughness covering by the layer, as well as (iv) the layer thickness, profiling, and actual chemical composition. One possibility for complex characterization of modified surfaces is Raman spectroscopy/microscopy, including variations like sum frequency generation (SFG) vibrational spectroscopy.^18^ For mixed polymer/surfactant layers on particular surfaces (polypropylene, nylon, and graphite), this method has been recently used and combined with contact angle determination.^18^ The combination of Raman and XPS spectroscopies was also applied for studying temperature changes in composite materials.^23^ XPS combined with Ar^+^ ion sputtering is one of the most important techniques for investigating the chemical composition and layered structure of thin-film coatings. This method enables not only the identification of elements present on the surface of a material but also the determination of their chemical states and compositional changes as a function of depth. A fundamental limitation of conventional XPS analysis is its relatively shallow information depth, typically a few nanometers.^24–28^ To obtain information from deeper regions of a sample, sequential sputtering of the surface with an argon ion (Ar^+^) beam is performed, followed by successive XPS measurements.^27,29,30^

For organic coatings, XPS combined with Ar^+^ sputtering provides valuable information on the distribution of elements such as carbon, oxygen, and nitrogen. The technique can be used to assess chemical homogeneity, and furthermore, it enables the investigation of changes in functional groups as a function of depth, making it particularly useful for studying surface modifications.^28^ However, it should be noted that depth profiling of organic materials using Ar^+^ ion sputtering may lead to damage of the chemical structure of the investigated layer. Ion bombardment can induce bond breaking, preferential sputtering of specific elements or molecular fragments, and alterations in the chemical states of atoms, resulting in artifacts within the obtained depth profiles.^30,31^ A good example of research dealing with the decomposition of organic structures during ion etching is the paper by Hofstetter and Vaynzof,^55^ which deals with the ion-beam etching (at various energies) of polymers. In that study, the authors demonstrate that significant changes occur in carbon bonds during etching—specifically deoxygenation—manifested by alterations in C1s signal intensities associated with oxygen-containing groups (C–O, C=O, or O–C=O), accompanied by an increase and broadening of the C=C and C–H carbon signals. In our measurements, ion etching could potentially have caused layer decomposition; however, confirming this would require a series of additional measurements using different Ar^+^ ion etching beam energies and subsequent analysis of the results, which was beyond the scope of the present study, and this topic could be a basis for a separate publication.

The characteristic feature of both glass and the organic layer is their microheterogeneity, connected with the presence of domains of varying chemical composition and crystallinity (for glass) and amphiphilic character of the material (for the coating material, comprising reactive surfactant and oligomer-type block copolymer). Moreover, only surfaces with low or moderate roughness may be analyzed by Raman microscopy and XPS spectroscopy with ion sputtering. Taking into consideration the aforementioned points, complex studies are possible by the combination of two different techniques: Raman microscopy and XPS spectroscopy with ion sputtering. Both techniques enable the analysis of chemical composition directly at the surface and up to a few microns in depth. The most significant differences are connected with the surface taken for data acquisition: a single “point” (with a resolution of a few dozens of nanometers) for Raman microscopy or an area of around 50–100 mm^2^ for XPS, as well as the reversibility of the measurement (possible for Raman microscopy and impossible for the previous layer after ion sputtering). Both techniques enable the detection of particular chemical bonds, the estimation of their abundance (e.g., surface concentration), as well as the analysis of elemental composition. For Raman spectroscopy, taking into account a very large number of particular spectra (924 for a sample of 12 μm × 11 μm × 6 μm), the K-means clustering algorithm has been utilized for the data analysis. To identify distinct patterns in these spectra (already compressed using PCA as described above), we first determined the optimal number of clusters. This was achieved using the elbow method, in which the inertia plot is examined to locate the point of diminishing returns.^56^ Based on this analysis, we selected 5 clusters as the most appropriate representation of the data.

Atomic force microscopy (AFM) was employed to evaluate the surface morphology and root-mean-square (RMS) roughness of a bare glass substrate and thin films deposited by spin coating, spray coating, and dip coating (scan area: 25.0 μm × 25.0 μm). The statistical standard deviation of the surface height profile obtained from an AFM image, namely, RMS, is highly sensitive to large peaks or deep valleys. The estimated RMS measurement uncertainty, calculated as the average of several images, did not exceed 3%. In our case, RMS values from height distribution histograms provide a quantitative metric for film quality and uniformity and reveal a clear correlation between deposition method and surface morphology. The bare glass substrate (Figure 1a) exhibits a high-density granular morphology with tightly packed nanoscale features and no large-scale defects, yielding an RMS roughness of 13.7 nm. The spin-coated film (Figure 1b) presents a dramatically smoother, nearly featureless surface with only sparse bright spots attributed to the glass surface or particulate contaminants, corresponding to the lowest RMS roughness of 3.6 nm and demonstrating effective planarization of the underlying substrate. In contrast, the spray-coated film (Figure 1c) displays the most irregular morphology, characterized by large-scale topographic variations, overlapping splat-like domains, and pronounced height fluctuations from incomplete droplet coalescence, which aligns with the highest RMS roughness of 39.8 nm. The dip-coated film (Figure 1d) shows an intermediate quality: a relatively continuous layer with horizontal withdrawal-direction streaks and discrete bright protrusions from meniscus instabilities or aggregates, consistent with its intermediate RMS value of 29.1 nm. Collectively, the images confirm the quantitative hierarchy of surface quality: spin coating (3.6 nm) ≪ bare glass (13.7 nm) < dip coating (29.1 nm) ≪ spray coating (39.8 nm), with smoother morphologies directly reflecting lower RMS values. Moreover, for additional visualization, 3D AFM images for all of the studied samples have been introduced in Figure S12 in the Supporting Information.

**Figure 1:**
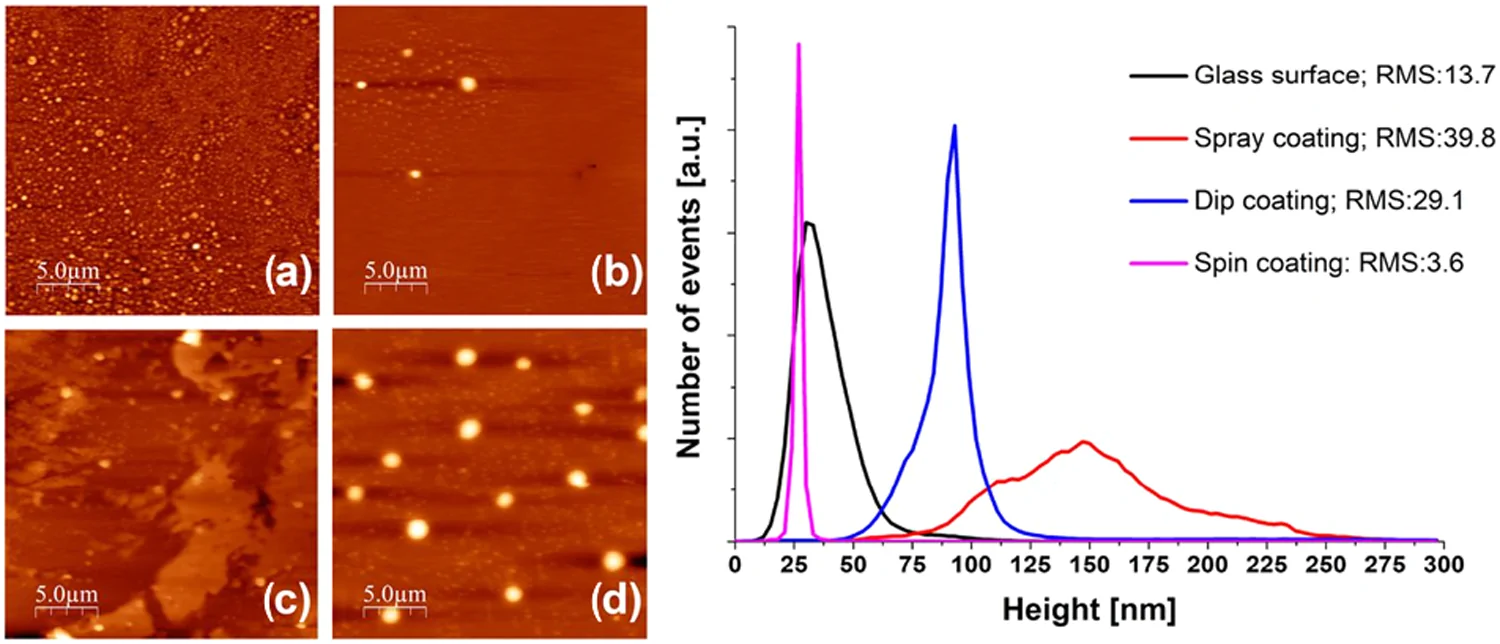
AFM images: clean glass (a), spin-coated film (b), spray-coated film (c), and dip-coated film (d) with height distribution histogram and RMS factors.

The AFM data show that the most valuable sample is 1A_0.5S_r2 (spin coating) because it has very uniformly distributed particles; XPS analysis with ion sputtering was performed on this sample. Moreover, pristine sample 1A_0.5S_2w2 (dip coating) was studied by XPS without ion sputtering due to detection of sp^2^ carbon bonds, indicating the presence of nonpolymerized carbosilane surfactant; in such a case, energetic Ar^+^ may initiate numerous reactions resulting in a total change of sample composition. The obtained XPS results with Ar^+^ ion sputtering for the investigated layers are summarized in Table 3.

**Table 3:** Atomic Concentrations in [%] of Individual Elements Identified in Sample 1A_0.5S_r2 (Spin Coating)

	FRESH	first	second	third	fourth	fifth	sixth	seventh	eighth	ninth
Si	19.7	18.1	19.3	22.6	19.4	21.8	23.6	23.7	25.2	24.5
C	52.2	44.1	39.8	34.7	34.8	30.0	27.5	25.8	23.3	23.8
N	1.9	1.6	1.8	0.8	2.1	1.8	1.5	1.1	1.3	1.0
O	25.1	35.3	37.3	40.3	42.1	45.5	45.9	47.9	49.5	49.6
Na	1.1	0.8	1.8	1.7	1.6	0.9	1.5	1.5	0.8	1.2

The actual atomic concentrations of the individual elements obtained in these measurements do not accurately reflect the nature of the layers; therefore, atomic Si/C ratios were determined as a measure of the layer presence on the sample. For this purpose, the ion source operation was calibrated using two types of samples: (i) a glass substrate coated with a gold layer onto which the target layer was deposited and (ii) the actual sample deposited on a glass substrate. As mentioned in the text, etching of layers deposited on a gold buffer layer was performed to estimate the etching rate, which was then used to etch the actual layer. Such a procedure is similar to those leading to estimation of the ion etching rate using a standard such as SiO_2_/Si.^37–39^ This procedure allowed the ion-gun operating parameters to be adjusted for interaction with the investigated layer. If the layer was etched too slowly, it would be impossible to see when the removal of deposited residual gases ends and when the actual etching of the layer begins, and also the whole process will be very stretched on the time scale. On the other hand, too fast etching would result in the removal of the entire applied layer in one ion etching process, without getting any information on layer composition.^57,58^ The calibration results are presented in Figure 2. For survey XPS spectra (spin-coating sample and calibration with a Au layer), see Figures S1 and S2, respectively.

**Figure 2:**
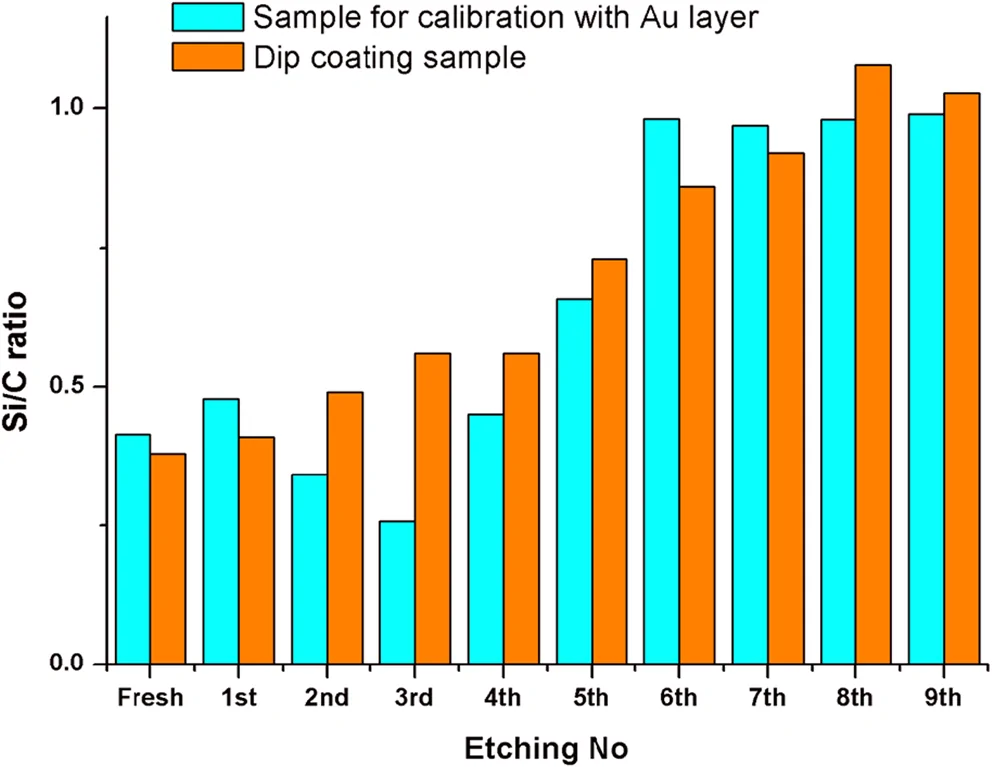
Atomic concentration ratios for layers deposited on the Au-coated substrate and bare glass (spin coating).

It can be noted that for the sample deposited on the Au layer, gold was detected at the fifth etching cycle, which, in relation to the main layer, resulted in the completion of the ion etching cycle on the sixth etch. The results of the concentrations of individual elements are presented in Table 4.

**Table 4:** Atomic Concentrations in [%] of Elements Present on the Surface with the Au Layer

	FRESH	first	second	third	fourth	fifth	sixth
Si	21.4	20.4	18.6	15.6	20.3	21.6	25.1
O	24.8	31.5	23.1	20.1	32.3	42.8	45.0
N	1.6	2.4	1.8	2.0	1.8	1.6	0.0
C	51.5	42.7	54.3	60.7	45.1	32.9	25.6
Cl	0.8	3.1	2.2	1.6	0.6	0.5	0.0
Au	0.0	0.0	0.0	0.0	0.0	0.5	4.4

It should be noted that during the conducted studies, in the C1s carbon region, besides the standard C–C, C–O, and C=O–C bonds, a strong Si–C bond at 283 eV^59^ was observed (Figure 3a), which was not detected for the sample prepared by the dip-coating method (Figure 3b). Some of the identified carbon bonds (especially C=O–C bonds) may originate from the air during sample transport. They may also originate from the ion etching process; however, since they are visible even on layers freshly introduced for XPS measurements (without ion etching), they are more likely residues from sample transfer in air.

**Figure 3:**
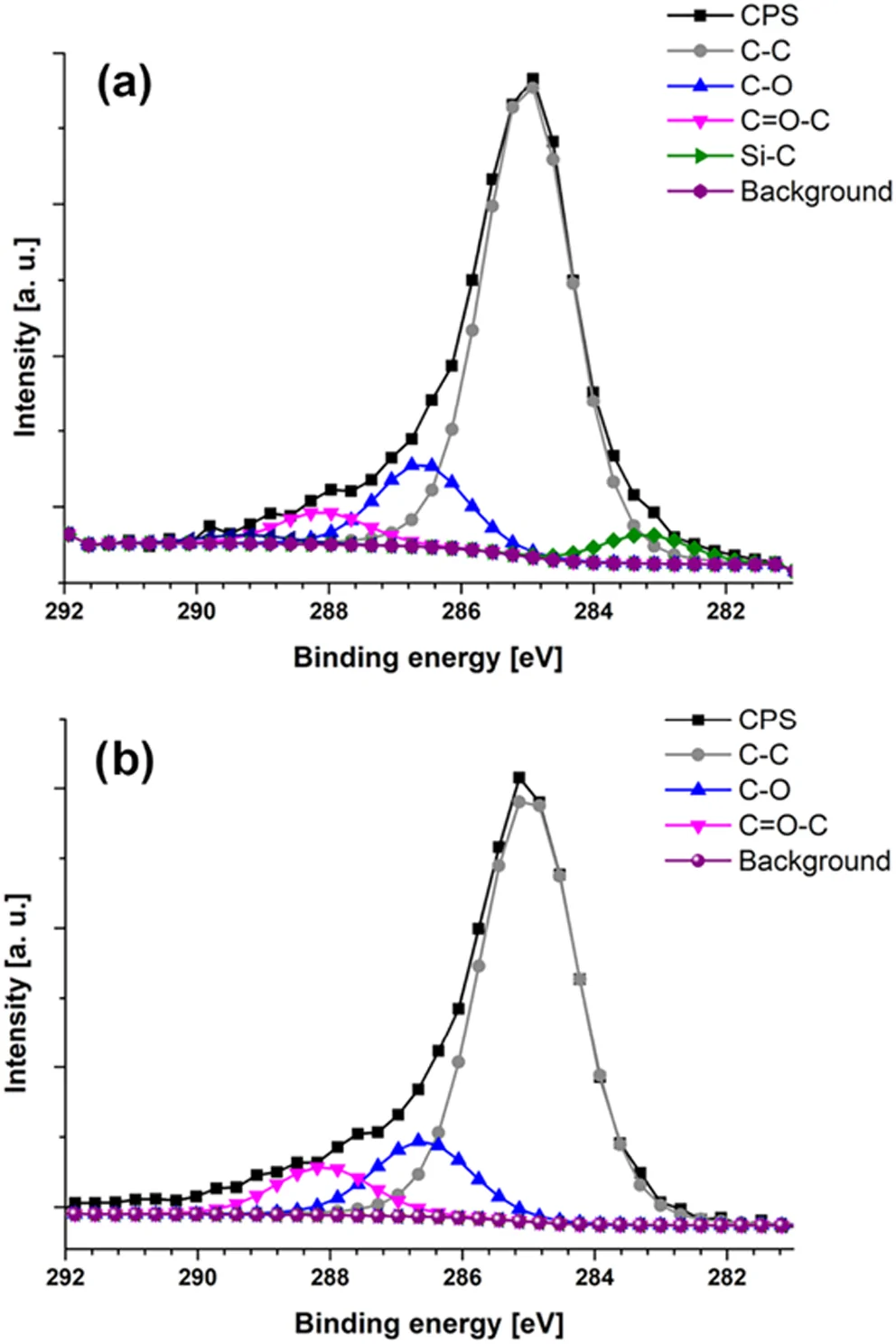
C 1s carbon region spectra for fresh samples, showing the presence of the Si–C bond for the spin-coating deposition method (a) and the dip-coating method (b).

XPS and ion sputtering, combined with compatible AFM microscopy, can provide unique data, confirming the layered structure of the studied systems. On the one hand, AFM images give us only information about surface topography—we can see the distribution of nanoparticles on the surface but without information about their chemical composition, especially whether the particles have a core–shell structure. On the other hand, XPS data combined with Ar^+^ ion sputtering can reveal the structure of nanoparticles, especially if they have a core–shell structure. First, ion sputtering removes residual deposits adsorbed on samples during transportation in air, and further ion sputtering cycles reveal the surface structure. The key factor of Ar^+^ ion sputtering is knowing the rate of material removal, so samples with a layer on the gold film were prepared and processed. From those measurements, we can conclude that after the fourth sputtering, we can see a signal from the gold layer, which means that almost all of the adsorbates were removed from the surface. Having this information, we knew the sputtering ratio, and after two sputtering cycles, we got chemical structure of a layer. Without AFM images, it would be very hard to determine whether it is a layered structure or a core−shell structure or 3D objects of the nanoscale features.

The results of clusterization for Raman microscopy measurements (see Table 5 and Figures 4 and 5, for samples obtained by spin coating (1A_0.5S_r2), and Figures S3–S6, for samples obtained by dip coating—1A_0.5S_1w1 and 1A_0.5S_2w2) clearly show that only spin coating enabled to prepare an explicit hydrophobic layer with a thickness of around 2–3 μm, sufficiently covering the cavities on the glass surface. Hence, we have confirmed the previous results of AFM, contact angle, and XPS measurements. On the other hand, any dip-coating approach resulted in the formation of a very thick hydrophobic layer with clearly visible domains of nonpolymerized reactive surfactants, especially within cavities. Spray-coating approaches led to a surface layer characterized by extreme roughness (see AFM image in Figure 1c), thus unsuitable for any Raman microscopy or XPS analyses. Moreover, the effectiveness of this approach toward surface hydrophobization was disputable due to limited homogeneity of the obtained layer (see Table 2).

**Figure 4:**
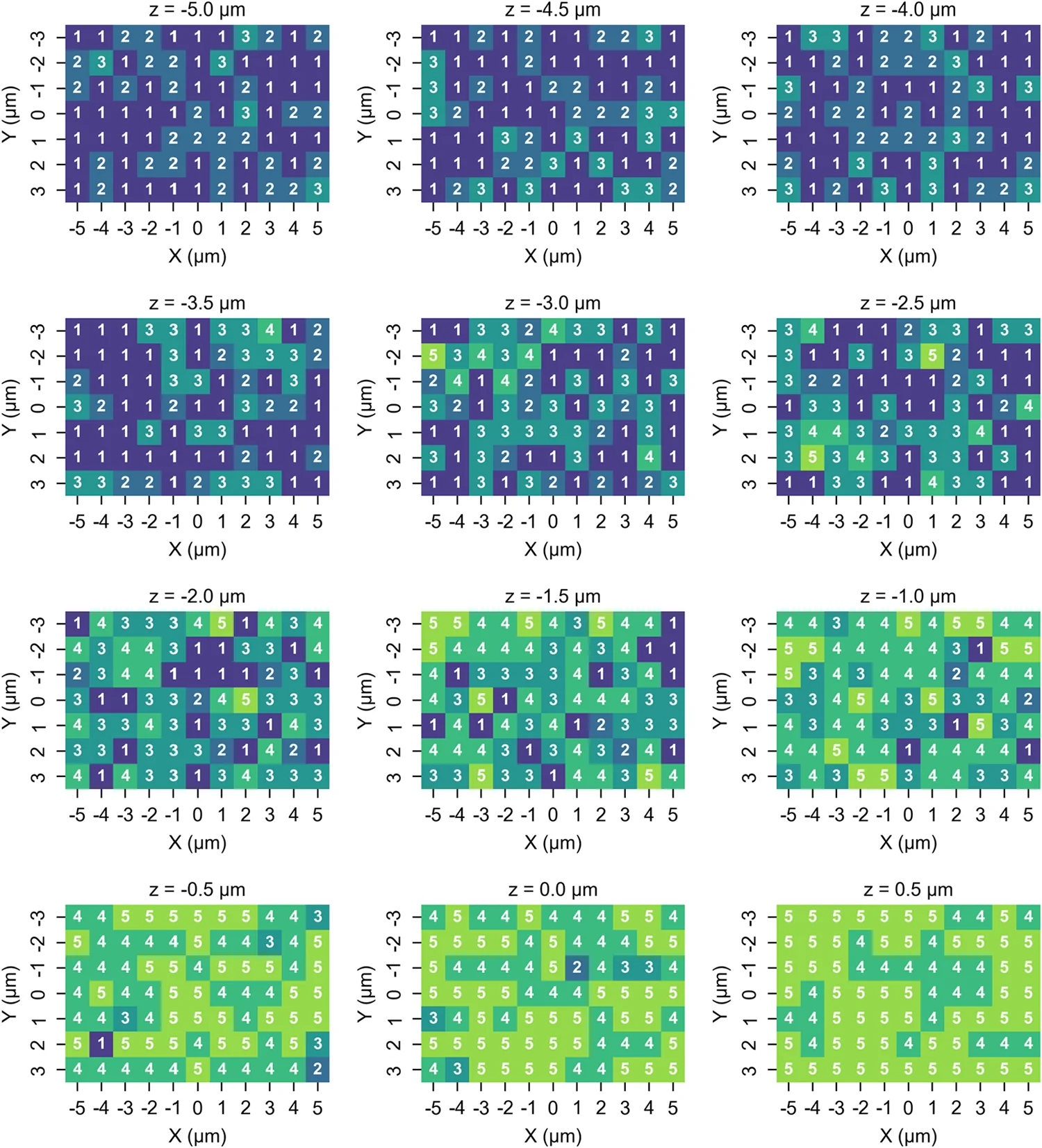
Heatmaps (Raman spectroscopy clusterization, spin coating, sample 1A_0.5S_r2 in Table 2) for particular depths.

**Figure 5:**
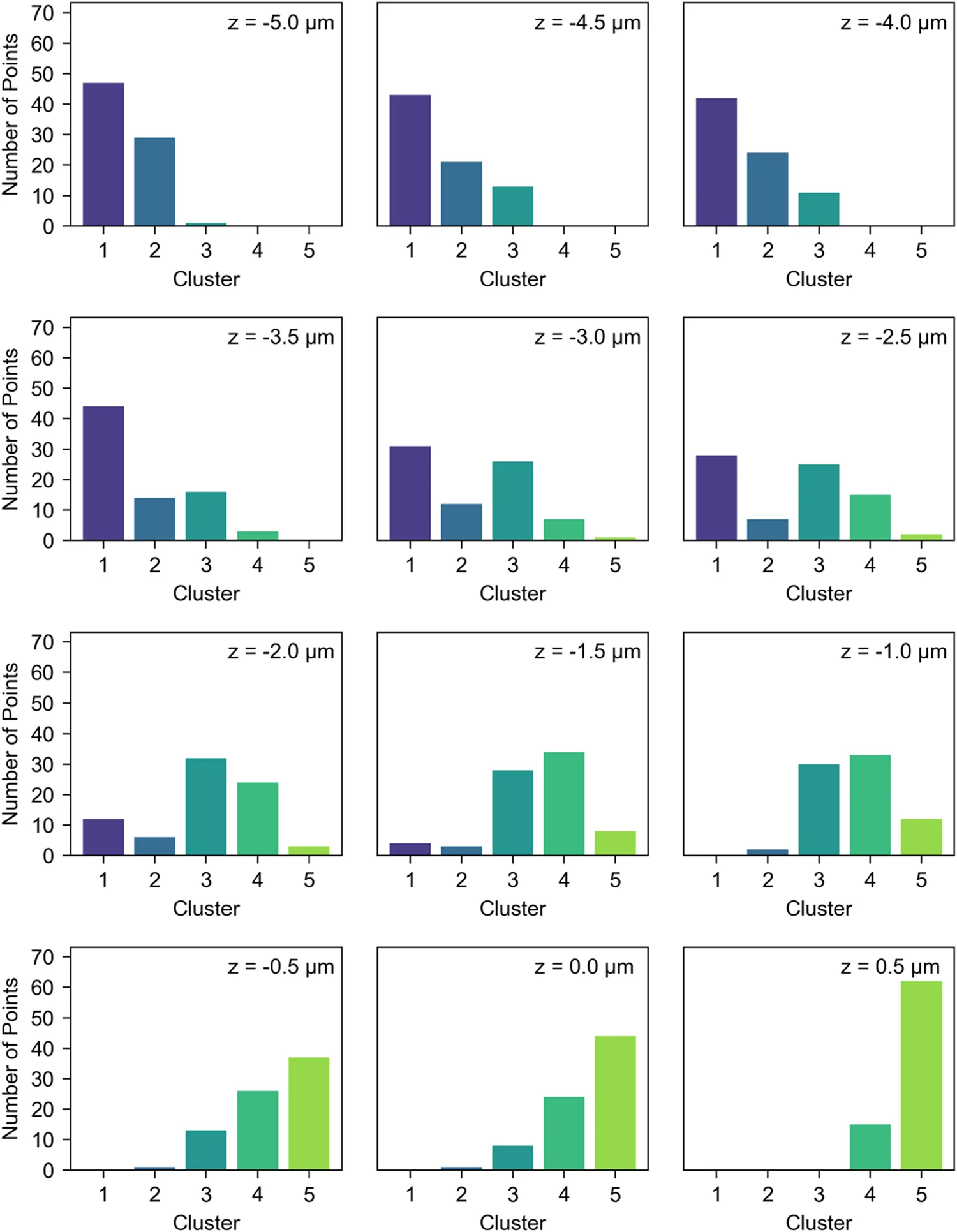
Histograms (Raman spectroscopy clusterization, spin coating, sample 1A_0.5S_r2 in Table 2) for particular depths.

**Table 5:**
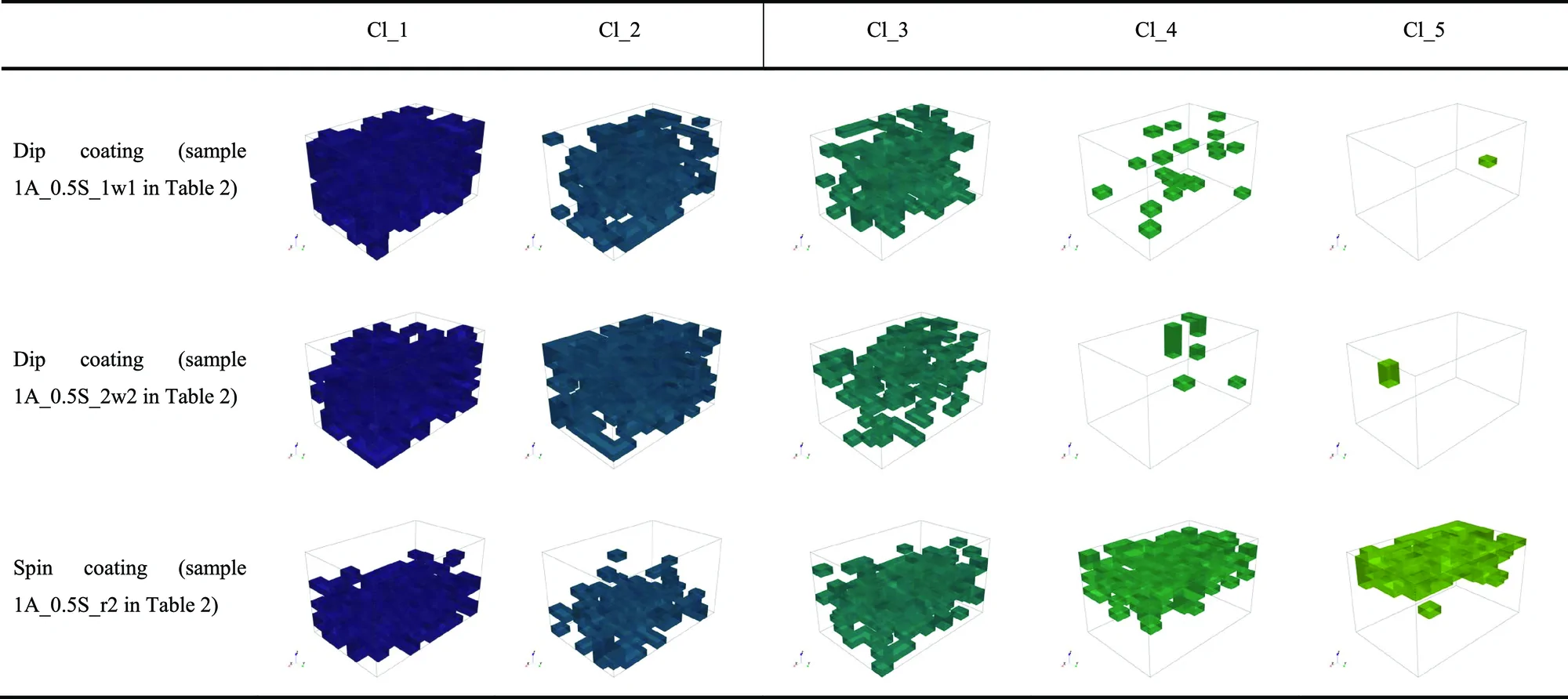
3D Visualization of Raman Microscopy Clusterization for the Selected Samples Obtained by Dip and Spin Coating

The clusterization efforts enabled deep insight into the analysis of Raman spectra (see Figures 6, S7, and S8 for the averaged spectra for particular clusters as well as Figures S9 and S10 for spectra of pure surfactant and glass surface, respectively). Raman spectrum peak at around 1600 cm^–1^ is characteristic of a double carbon–carbon bond; therefore, it is indicative of the presence of unreacted, nonpolymerized SK (see Figure S9). The presence of a double bond in the reactive silicone surfactant was also confirmed by FT-IR spectroscopy—clearly visible signals at ca. 1600 cm^–1^ and 920 cm^–1^ for C=C and =C–H bonds, respectively (see Figure S11). The disappearance of double-bond signals indicates consumption of vinyl groups, which may originate from both homopolymerization and Michael addition. The competitive ratio between these two pathways will be investigated in future work. In Figure 6, we can clearly see the differences between intensities of signals in ranges ca. 2250–2600 cm^–1^ and ca. 2900–3000 cm^–1^ for particular clusters. In general, for clusters linked to external organic layers (Cl_4 and Cl_5), the intensity of the broad signal in 2250–2600 cm^–1^ is lower when compared with clusters distinguished as glass components (Cl_1 and Cl_2), in contrast to the intensities of two peaks at ca. 2900 cm^–1^ and 2950 cm^–1^. Such behavior clearly shows the difference between those two groups of clusters, arose from organic matter constituting the external layer. The presence of two slightly different clusters for both the glass and the organic layer is, most probably, connected with microheterogeneity of both materials. On the other hand, Cl_3 may represent an interlayer between the glass and the organic layer or, more probably, some kind of glass subphases due to its presence in nearly all of the studied depths (excluding the outermost one). Such a hypothesis may be supported by the results of XPS studies (see Figure 3a), showing Si–C bonds that may be attributed to chemically attached terminal groups of AO (see Table 1). Reaction between alkoxyl motifs in amine-terminated oligosiloxane with O–O bridges and hydroxyl groups on the glass surface seems to be supported by the surfactant, enabling reduction of interfacial tension and better contact of the siloxane derivative with the substrate. Moreover, for the whole sample, there are no signs of unreacted silicone surfactant presence, indicating that during sample preparation, this compound completely polymerized or reacted (via Michael addition) with amine-terminated siloxane derivative. The analysis of samples obtained by dip coating revealed a lack of a clearly visible organic layer (see Table 5 and Figures S3–S6). On the other hand, those samples exhibited the presence of cavities or subdomains with unreacted silicone surfactant SK (clusters Cl_4 and, especially, Cl_5). In Figures S7 and S8, we can clearly see a very strong (for Cl_5) or weak/moderate (for Cl_4) signal at around 1600 cm^–1^, indicating the presence of a double carbon–carbon bond. Clusters Cl_1–Cl_3 may be distinguished as components/subphases in the glass structure. Raman microscopy studies clearly show that only spin-coating procedure may provide a stable hydrophobic layer on the glass surface, while any dip-coating approach results in separation of the oligomer and the surfactant, leading to only temporal hydrophobization by a thick, unattached layer of amine-terminated derivative. Moreover, for samples obtained by dip coating (1A_0.5S_2w2), XPS analyses revealed a lack of covalent bonds between the glass and the siloxane oligomer (Figure 3b), especially in the field of characteristic Si–C bonds, indicating the labile character of the hydrophobic layer. For better visualization, the reactions between SK and AO occurring during layer formation, as well as the differences in the structures of homogeneous and phase-separated layers obtained by spin and dip coating, respectively, are graphically illustrated in Figure 7.

**Figure 6:**
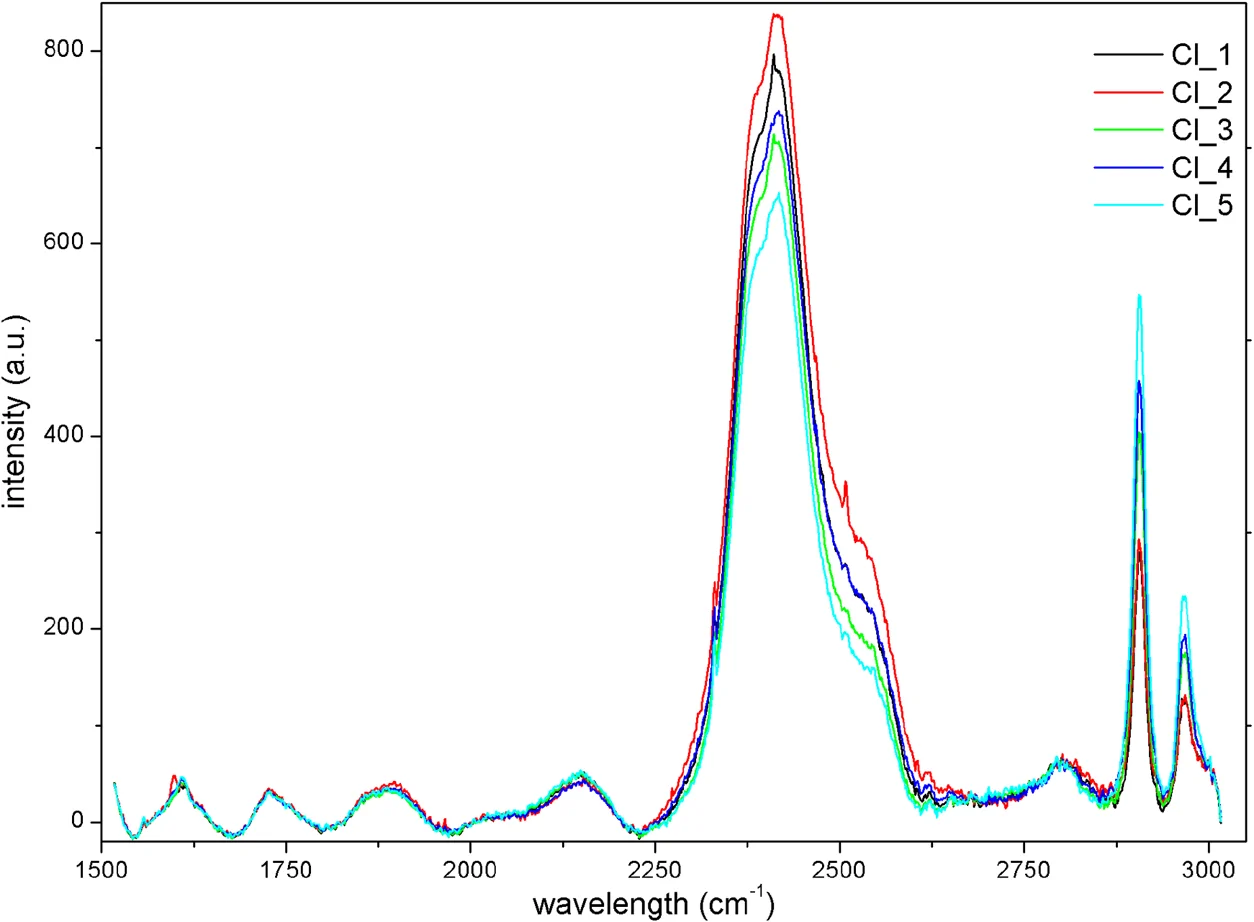
Averaged Raman spectra for particular clusters (Raman spectroscopy clusterization, spin coating, sample 1A_0.5S_r2 in Table 2).

**Figure 7:**
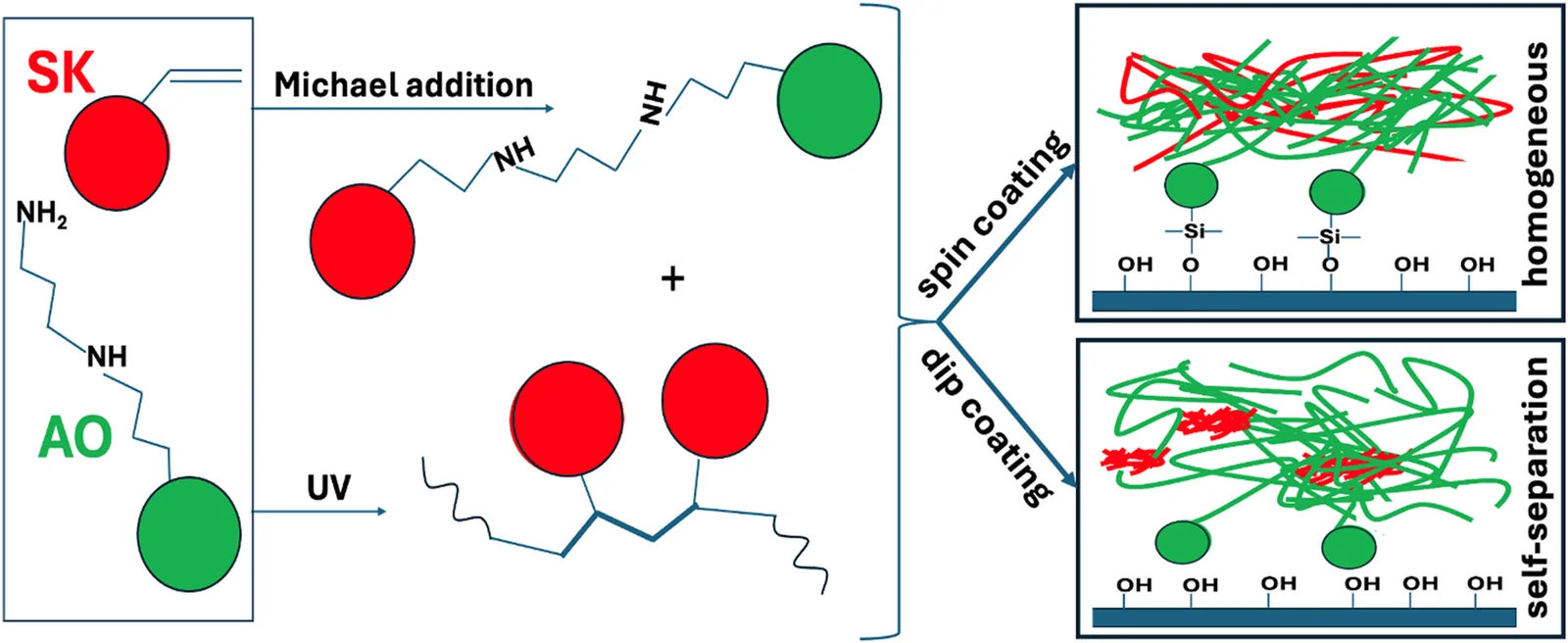
Reactions between SK and AO occurring during layer formation, as well as the differences in the structures of homogeneous and phase-separated layers obtained by spin- and dip-coating methods.

## Conclusions

The main aim of our studies—revealing the potential of a novel class of carbosilane reactive surfactants for glass hydrophobization—was reached by its combination with commercially available functional siloxanes with epoxy and amine motifs. Therefore, our SK surfactant has been carefully designed and synthesized in a multistep process, covering its main intended features: significantly limited susceptibility to undergo hydrolysis combined with the presence of a reactive (polymerizable) motif, compatibility with oligomeric siloxanes, and suitable reactivity within hydrophilic groups. Our investigations clearly show that epoxy-terminated oligodimethylsiloxane exhibits inferior ability to provide glass hydrophobization when compared with amine-terminated ones. For a mixture of amine-terminated oligodimethylsiloxane with our carbosilane reactive surfactant, the superior effectiveness of the coating process was obtained for spin coating—a stable layer with good coverage of surface unevenness, exhibiting a reduction of surface energy polar increment to less than around 5 mJ/m^2^. The stability of a such layer was also determined by treatment with water and isopropanol—both procedures provided no changes in its hydrophobic performance. For spray coating, the surface was very rough, and the contact angle measurements for determination of surface energy may be affected by this feature, although the superiority of amine-terminated oligodimethylsiloxane/carbosilane reactive surfactant system was clearly visible. An important part of our studies was the obtained layers’ characterization by AFM, Raman microscopy, and XPS spectroscopy with ion sputtering. Their results clearly show a lack of unreacted double bonds for the layer obtained by spin coating (no strong signal at ca. 1600 cm^–1^ in Raman spectra and no detectable amount of sp^2^ carbon) and chemical coupling of an amine silicone derivative by exchange of terminal alkoxyl motifs. For dip coating, a significant amount of underacted surfactant was found in the layer, while no additional covalent binding between the glass and the AO has been detected. It should be noted that the carbosilane reactive surfactant formed during the dip-coating procedure resulted in a regular pattern of deposits (possibly crystallites) on the glass surface.

Our studies revealed the usefulness of carbosilane reactive surfactants—a novel class of amphiphatic compounds—toward glass surface hydrophobization when combined with epoxy- and amine-functionalized oligodimethylsiloxanes. Careful analysis by XPS with Ar^+^ sputtering as well as Raman microscopy enabled us to find out the optimal methodology for the film deposition. Our study clearly showed the great potential of the utilized approach toward surface hydrophobization: mixing of a novel reactive surfactant of superior stability with commercial, functional silicone derivatives, supported by careful Hansen solubility parameter calculations, followed by appropriate AFM, XPS, and Raman studies for the layer characterization at both micro- and macroscale.

## Supplementary Material



## Notes

Survey XPS spectra of the analyzed layer and calibration system deposited on Au-covered glass; heatmaps and histograms for Raman microscopy clusterization (dip coating); averaged Raman spectra for particular clusters (dip coating); Raman and FT-IR spectra for reactive silicone surfactant and glass surface; 3D AFM images; coating stability experiments (PDF)
