# Supplementary material for: Custom-Designed Carbosilane-Based Reactive Surfactants for Surface Hydrophobization—AFM, Raman Microscopy, and XPS Investigations
Source: Langmuir. 2026 Oct 29;42(44):32901–17. doi: 10.1021/acs.langmuir.6c05105 (PMC13757964; doi:10.1021/acs.langmuir.6c05105)
Supplement: Supplementary file 1 [file la6c05105_si_001.pdf]

Supporting Information

Custom-designed carbosilane-based reactive surfactants for surface hydrophobization – AFM, Raman microscopy, and XPS investigations

Sylwia Ronka [a)*], Łukasz Lamch [b)], Maciej Kuchowicz [c)], Rafał Szukiewicz [c)], Przemysław Szklarz [d)], Krzysztof Gawarecki [e)]

[a)] Department of Polymer Engineering and Technology, Faculty of Chemistry, Wrocław University of Science and Technology, Wybrzeże Wyspiańskiego 27, 50-370 Wrocław, Poland

[b)] Department of Engineering and Technology of Chemical Processes, Faculty of Chemistry, Wrocław University of Science and Technology, Wybrzeże Wyspiańskiego 27, 50-370 Wrocław, Poland

[c)] Institute of Experimental Physics, Faculty of Physics, University of Wrocław, Maxa Borna 9, 50-204 Wroclaw, Poland

[d)] Faculty of Chemistry, University of Wrocław, F. Joliot-Curie 14, 50-383 Wrocław, Poland

[e)] Institute of Theoretical Physics, Faculty of Fundamental Problems of Technology, Wrocław University of Science and Technology, Wybrzeże Wyspiańskiego 27, 50-370 Wrocław, Poland

* Corresponding author.

Email address: sylwia.ronka@pwr.edu.pl (S. Ronka)

Table of contents Page

1. Survey XPS spectra of analyzed layer for the studied sample (1A_0.5S_r2 – see Table 2 in main body) and calibration system deposited on Au-covered glass S3

2. Heatmaps and histograms for Raman microscopy clusterization (dip coating) S5

3. Averaged Raman spectra for particular clusters (dip coating) S9

4. Raman and FT-IR spectra for reactive silicone surfactant and glass surface S11

5. 3D AFM images S12

6. Coating stability experiments S13

1. Survey XPS spectra of analyzed layer for the studied sample (1A_0.5S_r2 – see Table 2 in main body) and calibration system deposited on Au-covered glass

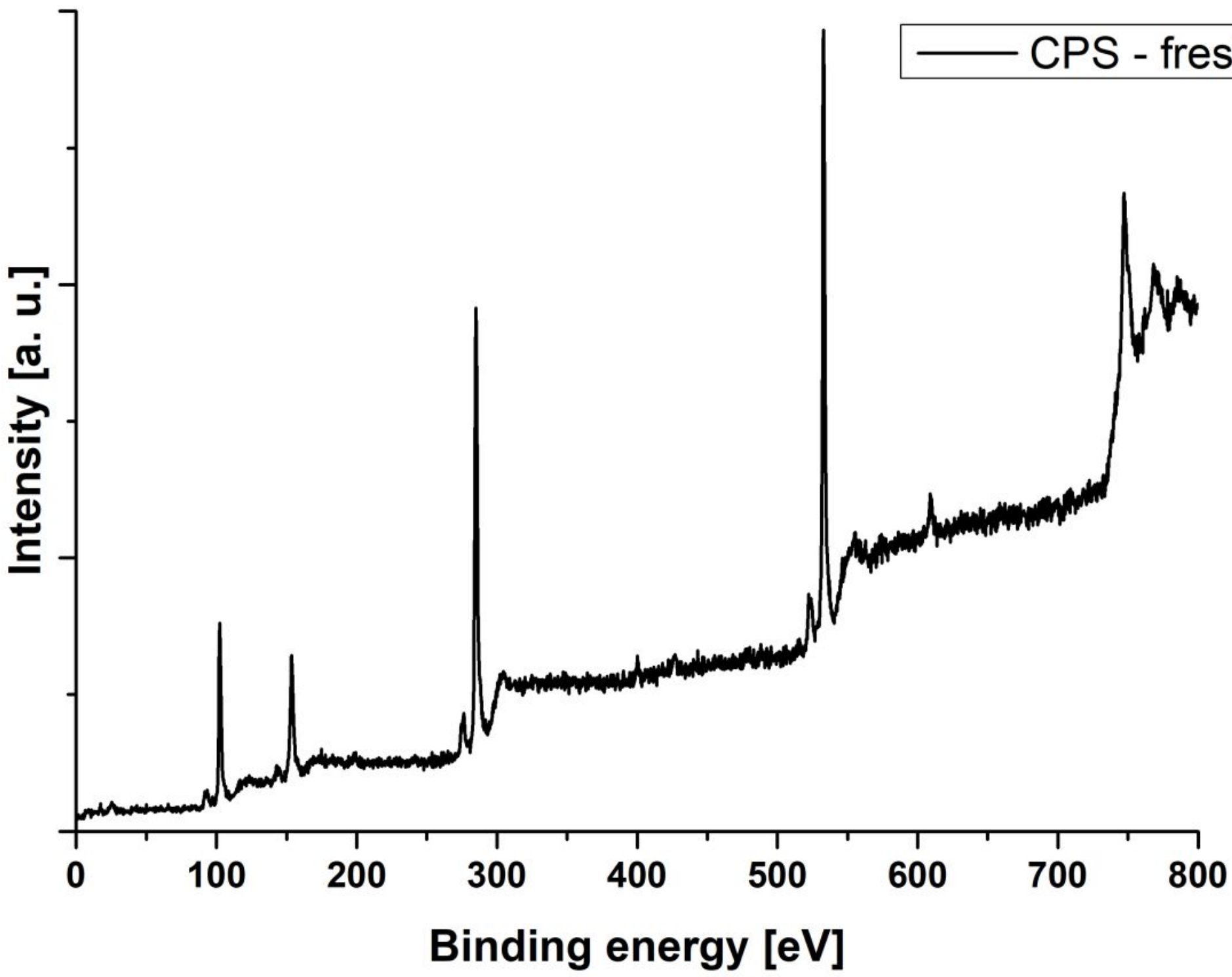

Figure S1. Survey spectra of analyzed layer after introducing into a UHV chamber (CPS – fresh).

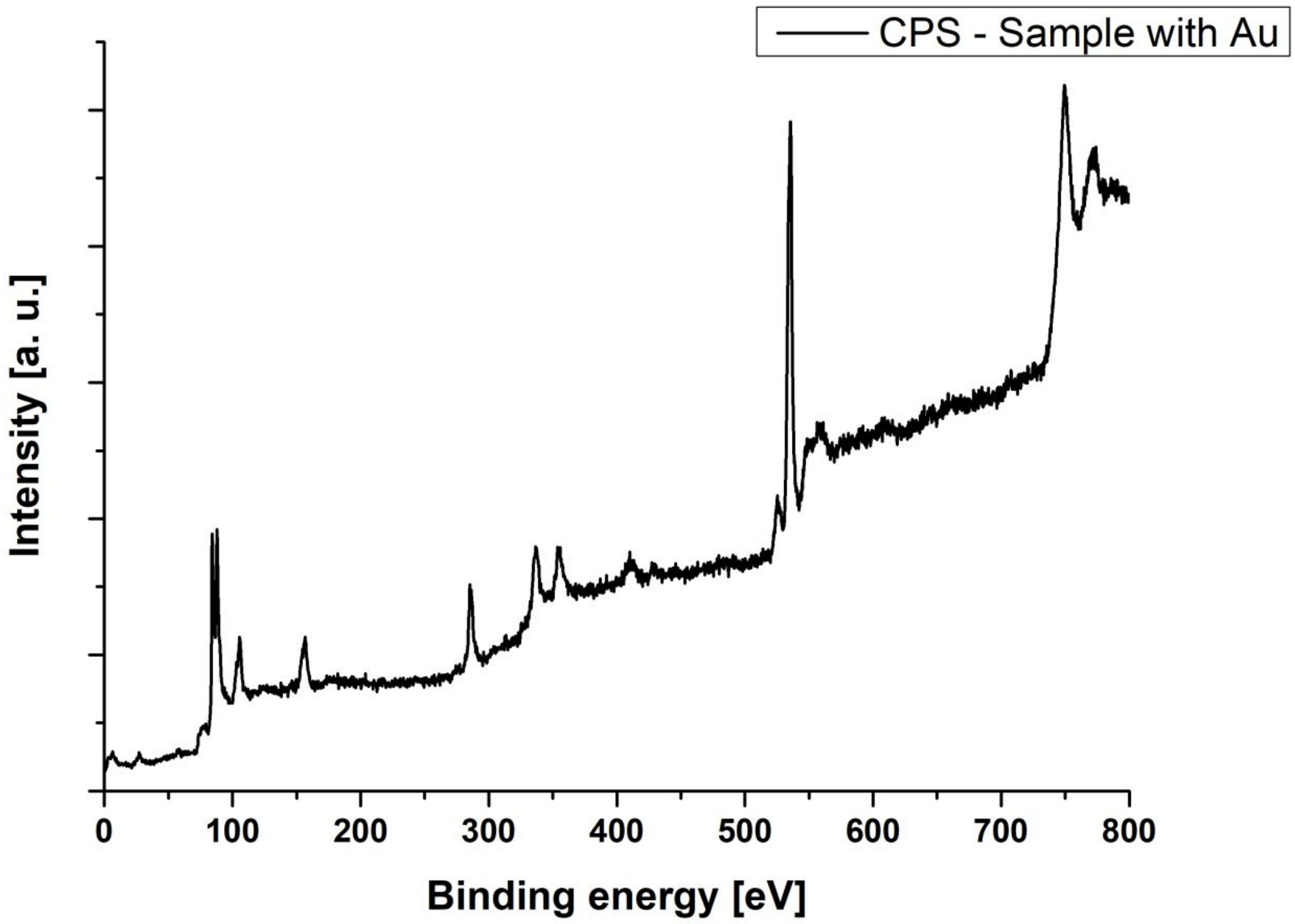

Figure S2. Survey spectra of analyzed layer deposited on Au after Ar^+ etching procedure (CPS - Sample with Au).

2. Heatmaps and histograms for Raman microscopy clusterization (dip coating)

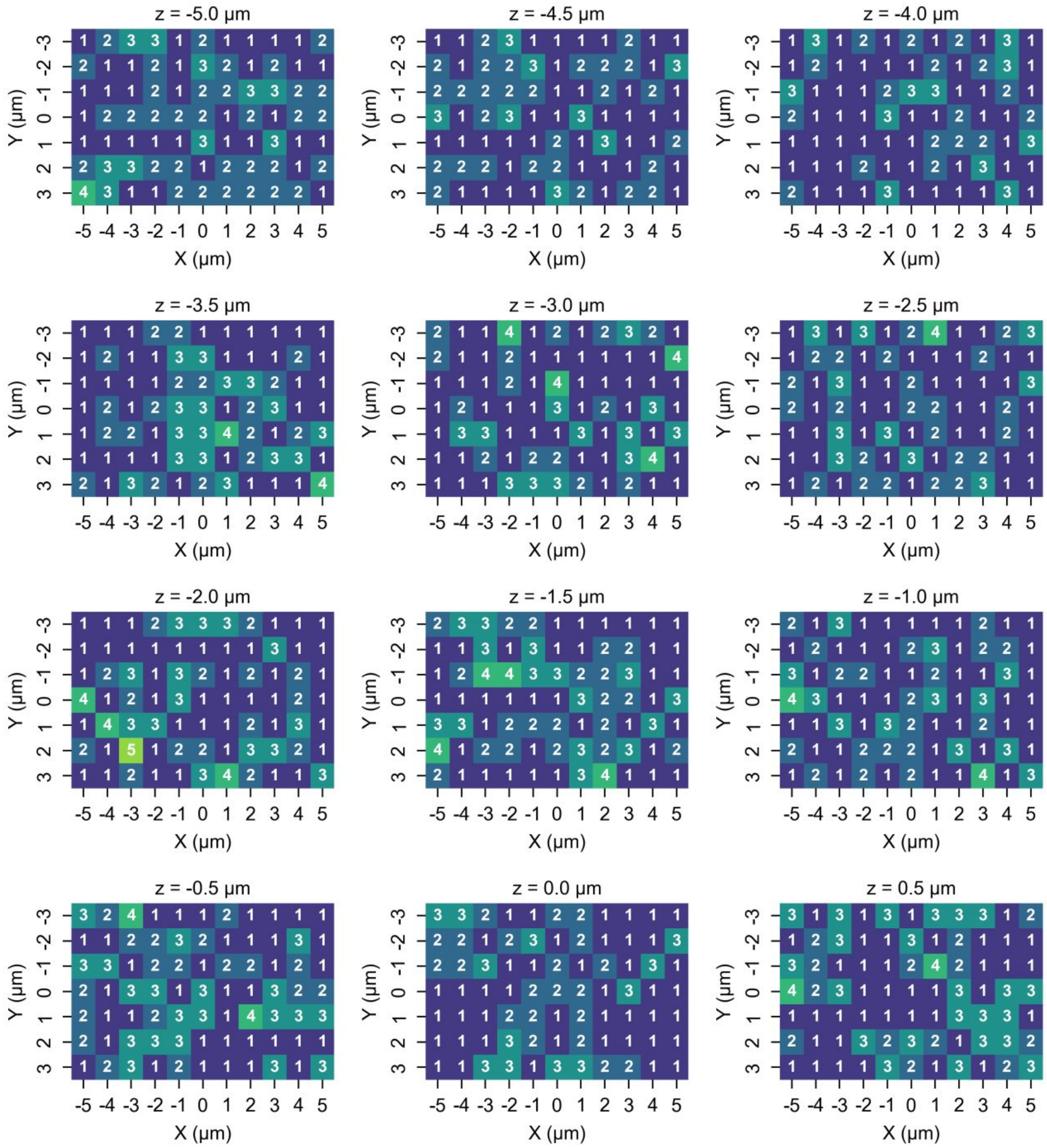

Figure S3. Heatmaps (Raman spectroscopy clusterization, dip coating, sample 1A_0.5S_1w1 in Table 2) for particular depths.

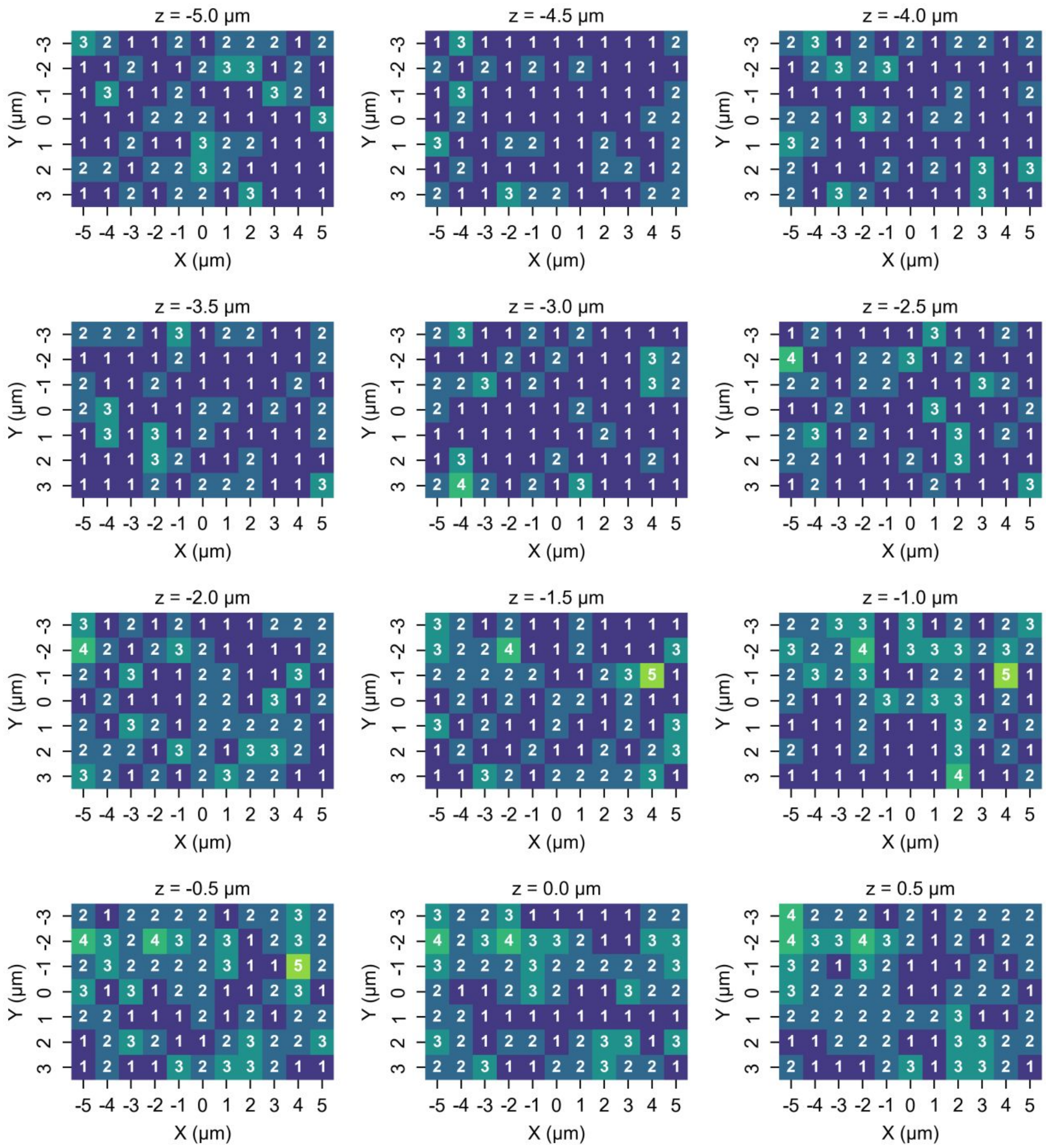

Figure S4. Heatmaps (Raman spectroscopy clusterization, dip coating, sample 1A_0.5S_2w2 in Table 2) for particular depths.

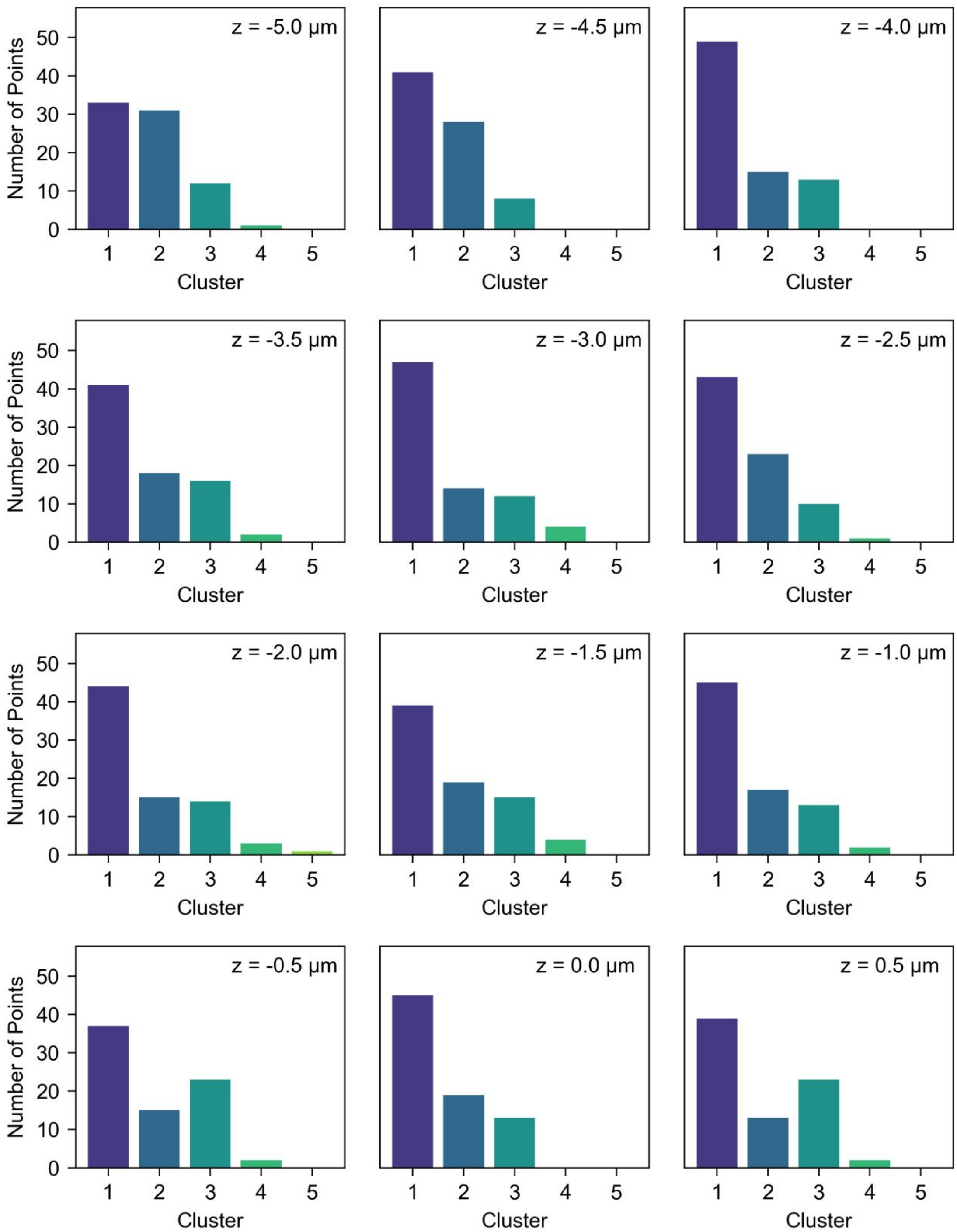

Figure S5. Histograms (Raman spectroscopy clusterization, dip coating, sample 1A_0.5S_1w1 in Table 2) for particular depths.

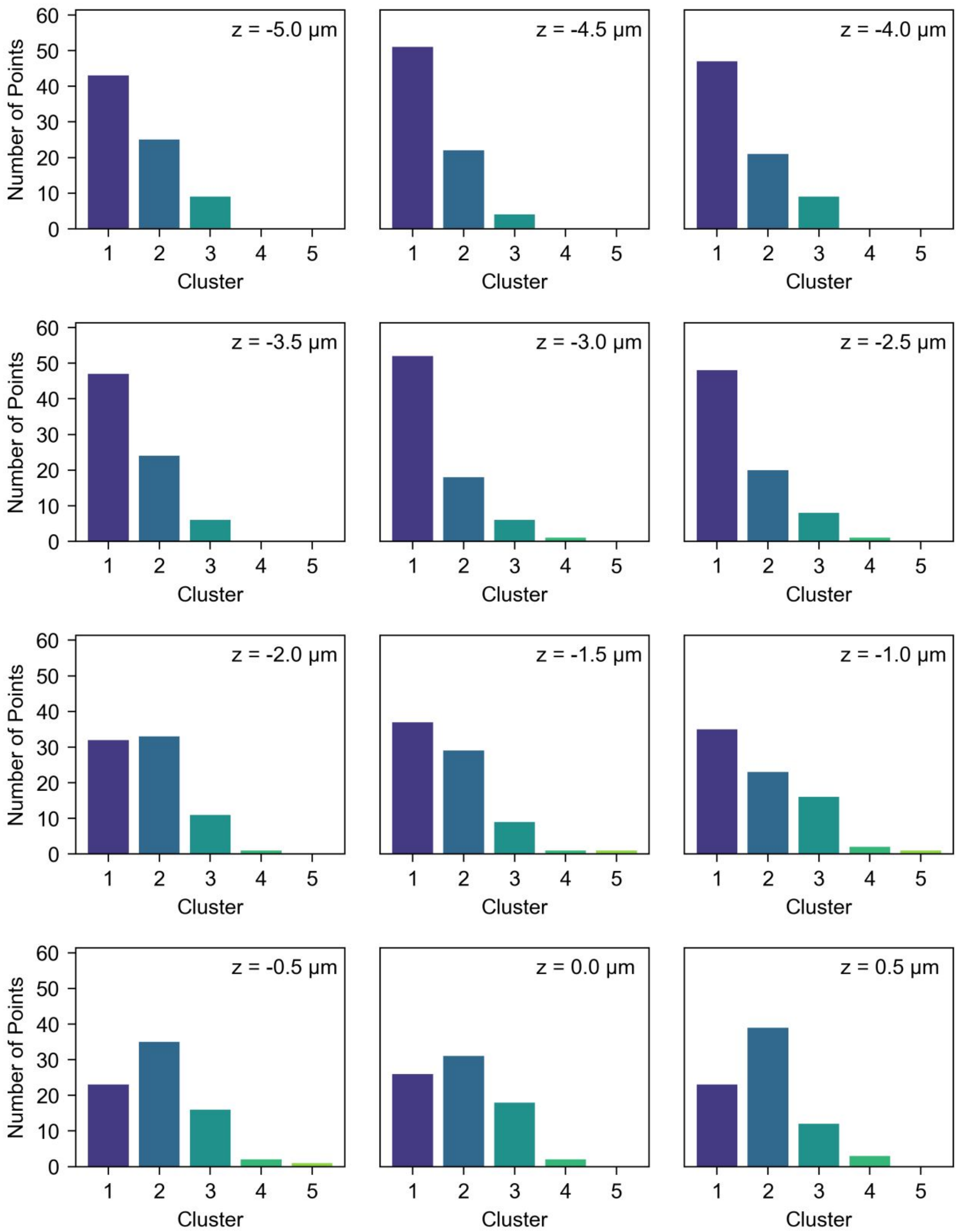

Figure S6. Histograms (Raman spectroscopy clusterization, dip coating, sample 1A_0.5S_2w2 in Table 2) for particular depths.

3. Averaged Raman spectra for particular clusters (dip coating)

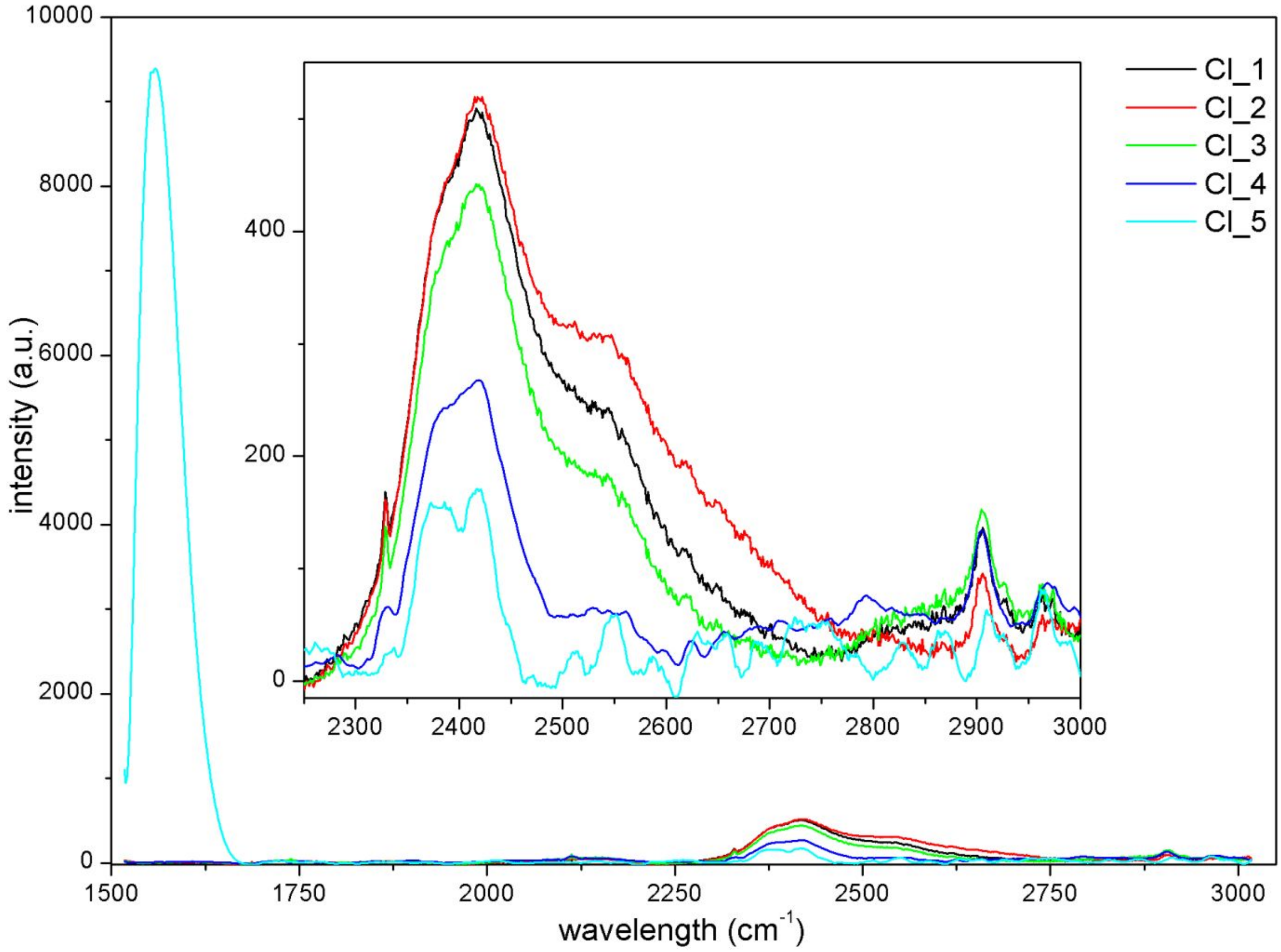

Figure S7. The averaged Raman spectra for particular clusters (Raman spectroscopy clusterization, spin coating, sample 1A_0.5S_1w1 in Table 2).

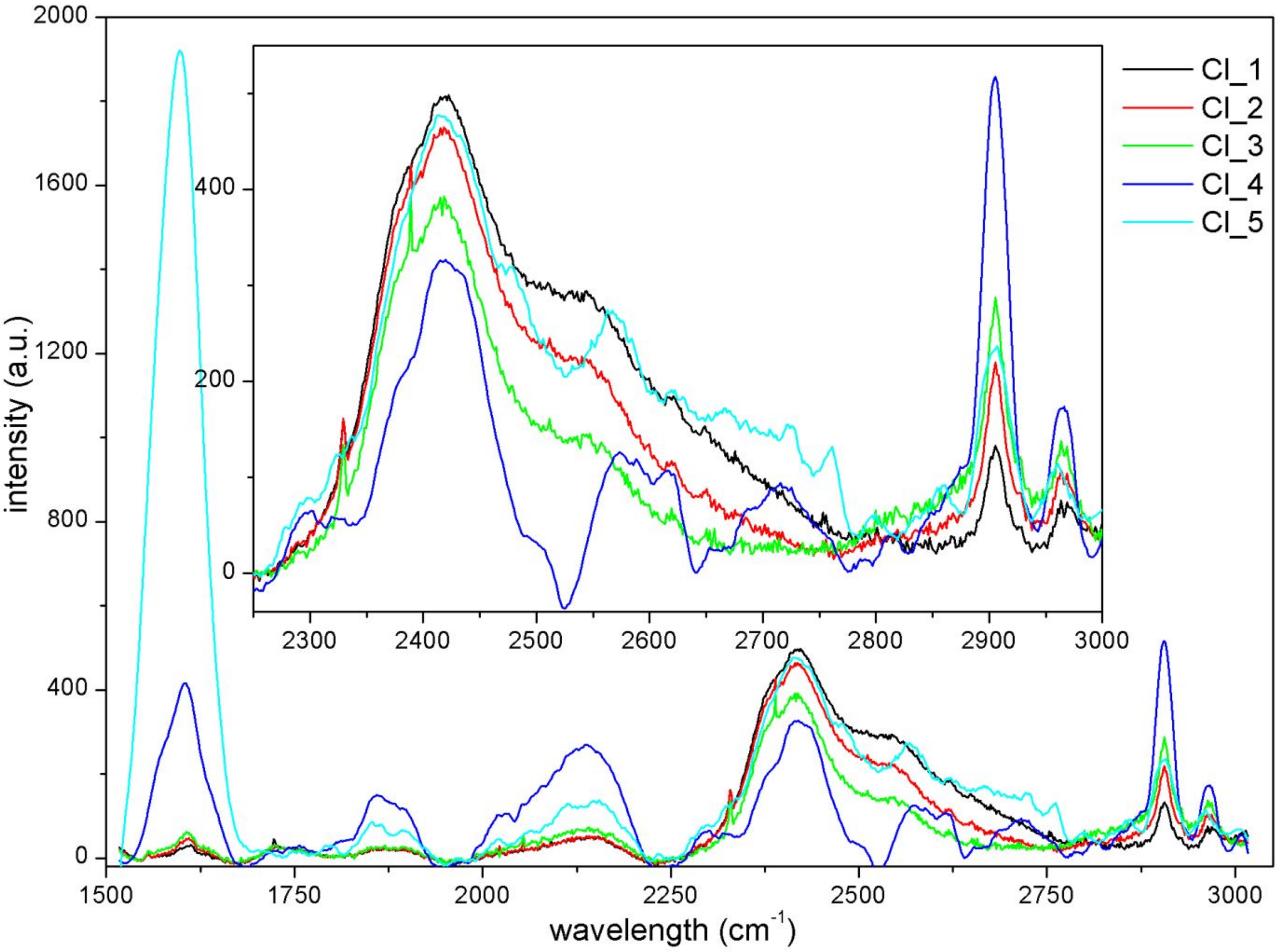

Figure S8. The averaged Raman spectra for particular clusters (Raman spectroscopy clusterization, spin coating, sample 1A_0.5S_2w2 in Table 2).

4. Raman and FT-IR spectra for reactive silicone surfactant and glass surface

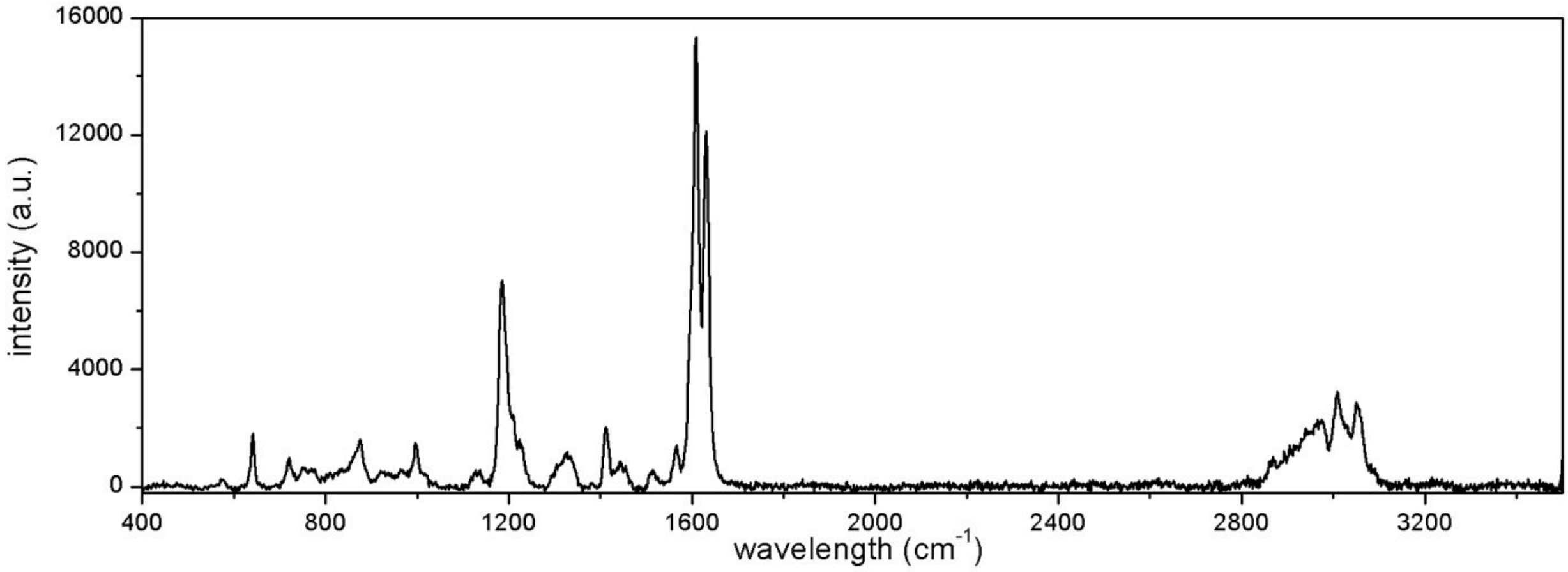

Figure S9. Raman spectrum for the studied reactive silicone surfactant.

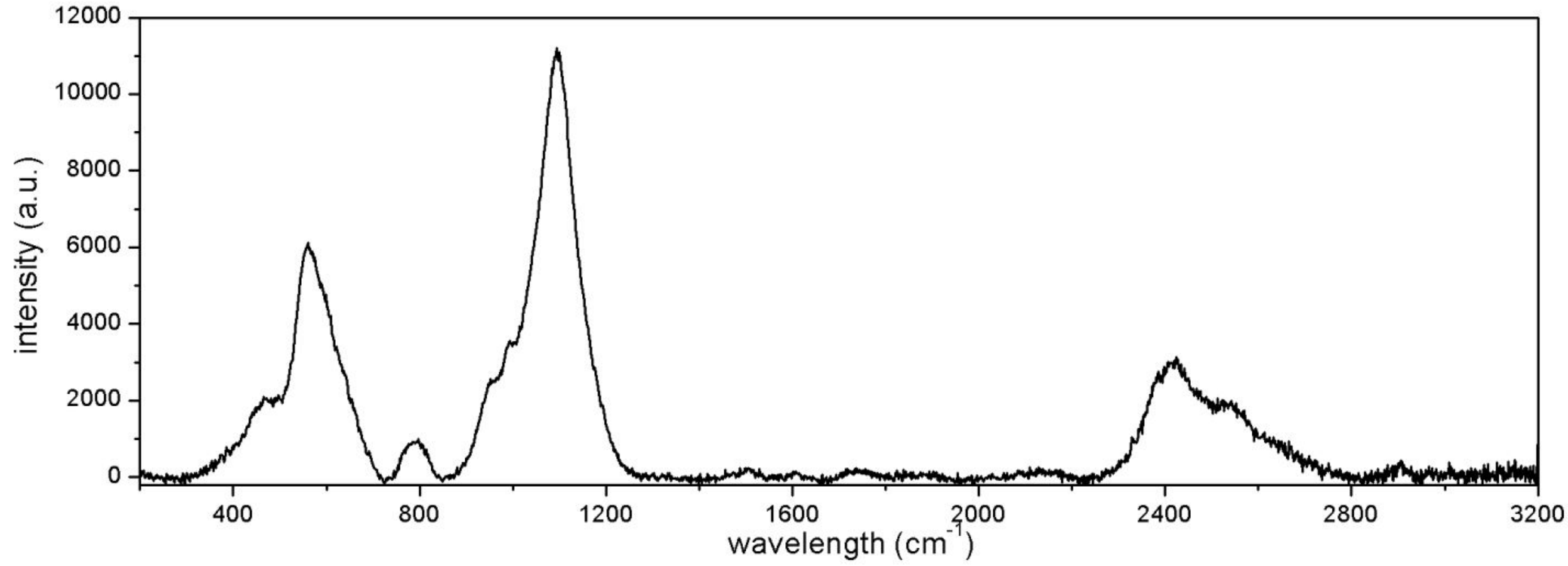

Figure S10. Raman spectrum for the pure glass surface.

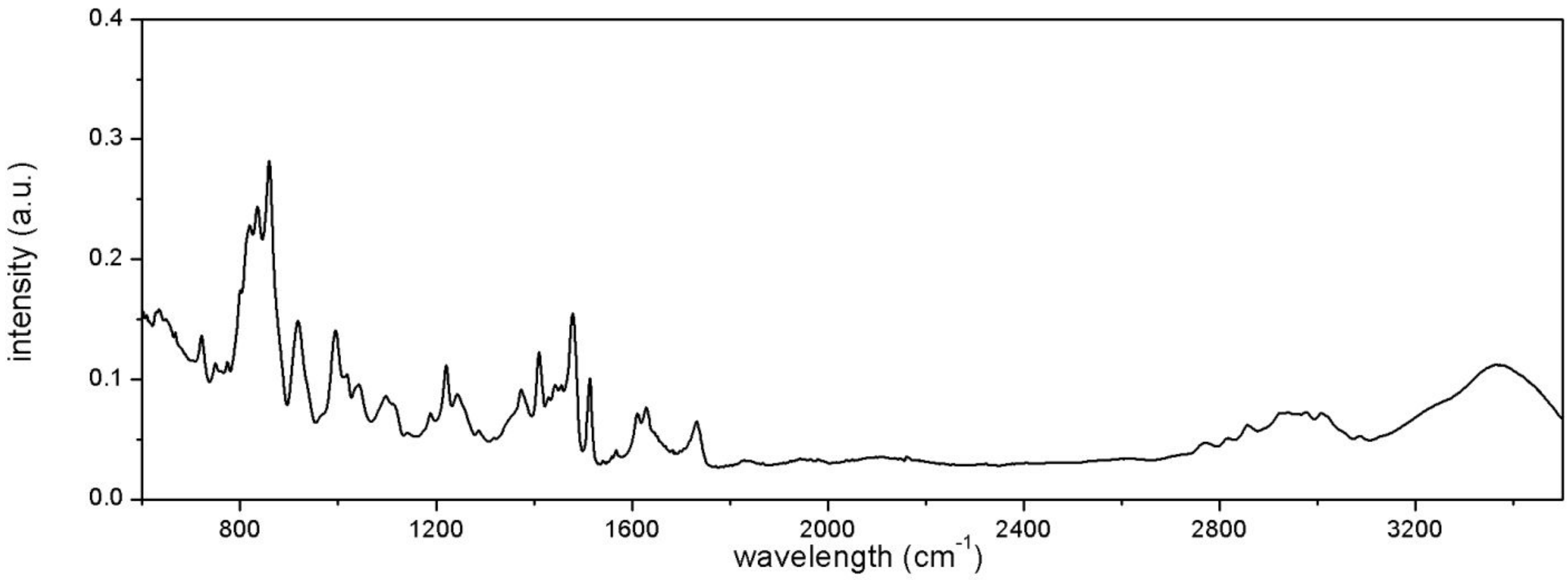

Figure S11. FT-IR spectrum for the studied reactive silicone surfactant.

5. 3D AFM images

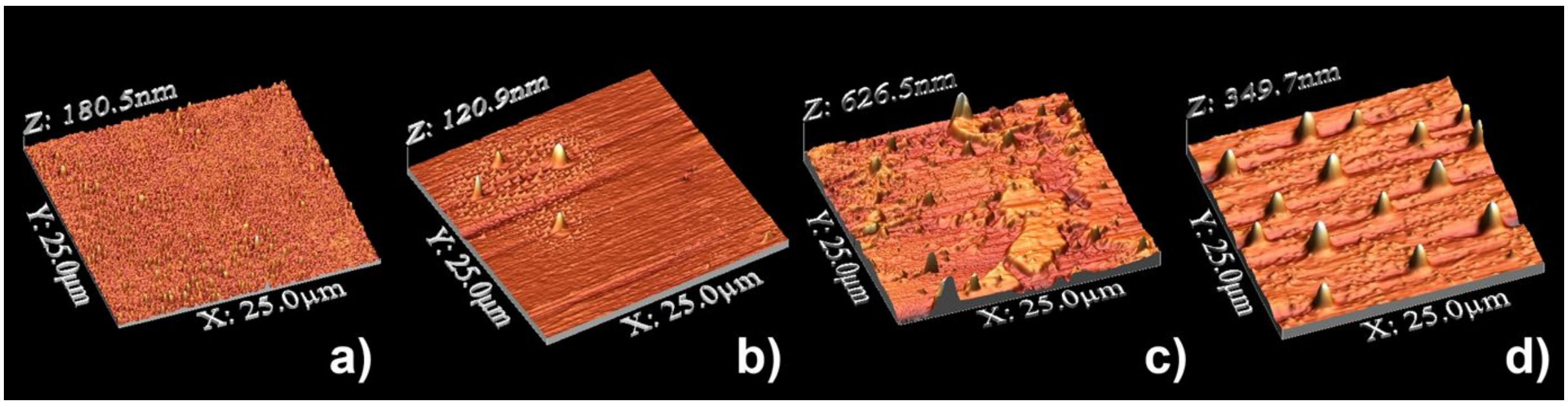

Figure S12. 3D AFM images for clean glass (a), spin-coated film (b), spray-coated film (c); dip-coated film (d).

6. Coating stability experiments

Table S1. The values of contact angles for water (θ_{H2O}) and diiodo-methane (θ_{DIM}), surface energies (γ_s) (disperse (γ_s^d) and polar (γ_s^p) components) for coating stability experiments.

Samples abbreviation*	description	γ_s [mJ/m^2]	γ_s^d [mJ/m^2]	γ_s^p [mJ/m^2]	θ_{H2O} [°]	θ_{DIM} [°]
**spray coating**						
1A_0.5S_d1	1% AO, 0.5% SK, d1	25.4 +/-0.4	20.2 +/-0.2	5.2 +/-0.2	90.6 +/-0.5	74.9 +/-0.4
1A_0.5S_d1_T	six-month coatings	21.4 +/-0.1	16.9 +/-0.1	4.5 +/-0.1	93.3 +/-0.1	81.1 +/-0.1
1A_0.5S_d1_H_2O	contacted with water	26.8 +/-0.2	21.2 +/-0.1	5.6 +/-0.1	86.9 +/-0.2	73.0 +/-0.2
1A_0.5S_d1_IPA	washed with isopropanol	22.6 +/-0.2	17.8 +/-0.1	4.8 +/-0.1	91.8 +/-0.2	79.4 +/-0.2
**spin coating****						
1A_0.5S_r2	1% AO, 0.5% SK, r2	26.2 +/-1.0	24.3 +/-0.5	1.9 +/-0.5	95.8 +/-2.3	67.5 +/-0.9
1A_0.5S_r2_T	six-month coatings	23.8 +/-0.4	20.0 +/-0.2	3.8 +/-0.3	92.7 +/-0.7	75.3 +/-0.4
1A_0.5S_r2_H_2O	contacted with water	24.0 +/-0.2	19.9 +/-0.1	4.1 +/-0.1	91.8 +/-0.3	75.4 +/-0.2
1A_0.5S_r2_IPA	washed with isopropanol	23.9 +/-0.2	21.1 +/-0.1	2.8 +/-0.1	95.3 +/-0.4	73.2 +/-0.2
**dip coating*****						
1A_0.5S_1w2	1% AO, 0.5% SK, 1w2	27.4 +/-0.5	22.2 +/-0.3	5.2 +/-0.2	87.3 +/-0.4	71.2 +/-0.6
1A_0.5S_1w2_T	six-month coatings	23.5 +/-0.4	18.9 +/-0.2	4.9 +/-0.2	91.2 +/-0.4	77.4 +/-0.2
1A_0.5S_1w2_H_2O	contacted with water	24.1 +/-0.2	20.5 +/-0.1	3.6 +/-0.1	92.8 +/-0.2	74.3 +/-0.2
1A_0.5S_1w2_IPA	washed with isopropanol	28.0 +/-0.2	19.8 +/-0.1	8.2 +/-0.1	82.4 +/-0.2	75.6 +/-0.1

Glass

Samples abbreviation	description	γ_s [mJ/m^2]	γ_s^d [mJ/m^2]	γ_s^p [mJ/m^2]	θ_{H2O} [°]	θ_{DIM} [°]
G_UV	Glass UV	56.9 +/-0.4	32.4 +/-0.2	24.6 +/-0.2	45.4 +/-0.4	53.3 +/-0.3
G_pure	Glass pure	48.2 +/-0.5	25.2 +/-0.2	24.0 +/-0.3	54.2 +/-0.4	65.9 +/-0.3

* sample d1– distance between the head and the glass surface: 60 cm;
** sample r2 – rotation speed: 1500 rpm; spinning time: 20 s;
*** sample 1w2 – withdrawal speed: 0.2 mm/s; number of dip cycles: 1.
